# Use of human predictive patch test (HPPT) data for the classification of skin sensitization hazard and potency

**DOI:** 10.1007/s00204-023-03656-4

**Published:** 2024-03-14

**Authors:** Matthias Herzler, Jaleh Abedini, David G. Allen, Dori Germolec, John Gordon, Hon-Sum Ko, Joanna Matheson, Emily Reinke, Judy Strickland, Hermann-Josef Thierse, Kim To, James Truax, Jens T. Vanselow, Nicole Kleinstreuer

**Affiliations:** 1 German Federal Institute for Risk Assessment (BfR), Berlin, Germany; 2 Inotiv, Inc., Morrisville, NC USA; 3 Systems Toxicology Branch, Division of Translational Toxicology, National Institute of Environmental Health Sciences (NIEHS), National Institutes of Health (NIH), Research Triangle Park, NC USA; 4 United States Consumer Product Safety Commission, Rockville, MD USA; 5 United States Food and Drug Administration, Silver Spring, MD USA; 6 National Toxicology Program Interagency Center for the Evaluation of Alternative Toxicological Methods (NICEATM), Predictive Toxicology Branch, Division of Translational Toxicology, NIEHS/NIH, Research Triangle Park, NC USA

**Keywords:** GHS, Skin sensitization, HMT, HRIPT, Weight of evidence

## Abstract

Since the 1940s, patch tests in healthy volunteers (Human Predictive Patch Tests, HPPTs) have been used to identify chemicals that cause skin sensitization in humans. Recently, we reported the results of a major curation effort to support the development of OECD Guideline 497 on Defined Approaches (DAs) for skin sensitization (OECD in Guideline No. 497: Defined Approaches on Skin Sensitisation, 2021a. 10.1787/b92879a4-en). In the course of this work, we compiled and published a database of 2277 HPPT results for 1366 unique test substances (Strickland et al. in Arch Toxicol 97:2825–2837, 2023. 10.1007/s00204-023-03530-3). Here we report a detailed analysis of the value of HPPT data for classification of chemicals as skin sensitizers under the United Nations’ Globally Harmonized System of Classification and Labelling of Chemicals (GHS). As a result, we propose the dose per skin area (DSA) used for classification by the GHS to be replaced by or complemented with a dose descriptor that may better reflect sensitization incidence [e.g., the DSA causing induction of sensitization in one individual (DSA1+) or the DSA leading to an incidence of induction in 5% of the tested individuals (DSA05)]. We also propose standardized concepts and workflows for assessing individual HPPT results, for integrating multiple HPPT results and for using them in concert with Local Lymph Node Assay (LLNA) data in a weight of evidence (WoE) assessment. Overall, our findings show that HPPT results are often not sufficient for deriving unambiguous classifications on their own. However, where they are, the resulting classifications are reliable and reproducible and can be integrated well with those from other skin sensitization data, such as the LLNA.

**Supplementary Information:**

The online version contains supplementary material available at 10.1007/s00204-023-03656-4.

## Introduction

### History of the project

In 2021, the Organization for Economic Co-operation and Development (OECD) published Test Guideline (TG) 497 on Defined Approaches for Skin Sensitization (OECD 2021a). This project established, for the first time, a testing and assessment strategy for skin sensitization hazard and potency prediction without the need for in vivo testing. The primary objective of this project was to validate the Defined Approaches (DAs) under examination for use in classification and labelling for skin sensitization under the United Nations’ "Globally Harmonized System of Classification and Labelling of Chemicals (GHS)” (UN 2023).

As a prerequisite for this work, the OECD expert group preparing TG 497 established a reliable set of reference classifications using two types of reference data: Local Lymph Node Assay (LLNA) and Human Predictive Patch Test (HPPT) data. Two dedicated sub-groups of experts were formed—an LLNA Sub-Group (LSG) and a Human Data Sub-Group (HDSG). These sub-groups reviewed the available data for a reference set of 196 substances to:

- eliminate errors in the data as far as possible,
- describe the variability and uncertainty associated with these data,
- explore their potential as well as limitations for classifying chemicals for skin sensitization, and
- use these data—where feasible—to classify the reference substances with respect to their skin sensitization potential according to the criteria and guidance values of the GHS in its current version.

A full account of the work performed by the two groups can be found in relevant OECD reports (OECD 2021b, c). The HDSG compiled and curated a database of 2277 individual HPPT results from the published literature. This work is described in more detail in another publication (Strickland et al. 2023). In the HDSG report (OECD 2021b), variability and uncertainty of the HPPT data were characterized to some degree for the subset of reference chemicals included in TG 497.

In the course of the work described above, it became obvious that the current GHS criteria and guidance values for classification, which use a single cut-off value for distinguishing strong (GHS sub-category 1A, Skin Sens. 1A) from other (GHS sub-category 1B, Skin Sens. 1B) sensitizers, have certain limitations. Specifically, as detailed below, the current practice of setting the cut-off for distinction between Skin Sens. 1A and 1B at a fixed dose per skin area (DSA) of 500 µg/cm^2^, without accounting for the relative fraction of sensitized test subjects, is prone to underrepresenting skin sensitization potency and is not equipped to deal with borderline cases.

Moreover, although the GHS recommends to users, where applicable, to integrate HPPT results with other evidence in a weight of evidence (WoE) approach for classification, it does not provide practical guidance on how to weigh HPPT results against results from other test or non-test methods suitable for the classification of skin sensitizers. In this article, therefore, we propose revised criteria for the classification of chemicals for their skin sensitization potential based on HPPT data and provide advice for their integration into an overall WoE assessment of the skin sensitization data available for a given chemical.

### Current GHS classification criteria for HPPT data

The GHS Rev. 10 provides criteria for classifying substances or mixtures^1^ as skin sensitizers based on human data, standard animal data, defined approaches, *in chemico*/in vitro data or data from non-test methods (UN 2023). With respect to human data, a substance (or mixture) is classified as a skin sensitizer (GHS Skin Sens. 1) “*if there is evidence in humans that the substance can lead to sensitization by skin contact in a substantial number of persons*”. Human evidence suitable in this regard can include positive results from Human Maximization Test (HMT) or Human Repeat Insult Patch Test (HRIPT) data, diagnostic patch test data or other epidemiological evidence. The GHS defines the way in which positive HPPT results can be used to classify chemicals as sensitizers and, where possible, to sub-categorize them for their sensitization potential based on the DSA (in µg/cm^2^) used for the induction step in such experiments. However, and different than for other tests for skin sensitization, the GHS does not define what constitutes a positive HPPT result. For the purpose of this work, we considered an HPPT result as “positive”, if sensitization of at least one test subject was reported as the consequence of exposure to the test item.

According to the GHS, if a positive result is obtained at an induction DSA ≤ 500 µg/cm^2^, the substance can be classified as Skin Sens. 1A, while, if the induction DSA is > 500 µg/cm^2^_,_ it can be classified as Skin Sens. 1B. If the DSA for a positive result (≥ 1 sensitized test subject) is unknown, sub-categorization is not possible based on this test result and the substance is classified as a Category 1 sensitizer (Skin Sens. 1).

Usually, each HPPT is performed using only one test concentration for induction. Therefore, under the GHS scheme, in many cases one cannot be certain whether a test at a different concentration would not have resulted in a stricter classification [i.e., Skin Sens. vs. Not Classified (NC)] or sub-categorization (i.e., Skin Sens. 1A instead of Skin Sens. 1B). The following scenarios can be distinguished:

- *Positive HPPT results obtained using a single induction DSA* ≤ 500 µg/cm^2^ unambiguously result in classification as Skin Sens. 1A.
- *Negative test results obtained at an induction concentration of *100% unambiguously result in no classification (NC). The same holds if the test concentration used was < 100%, but represents the highest achievable concentration (e.g., for technical reasons such as limited solubility or because of known local or even systemic toxicity at higher concentrations; however, such information is rarely available with the mostly historical HPPT data).
- *Positive HPPT results obtained using a single induction DSA* > 500 µg/cm^2^ can be used for classification as Skin Sens. 1B, but classification as Skin Sens. 1A cannot be ruled out with certainty, because a lower test concentration may also have produced a positive result. Such single test results therefore can be interpreted as pointing at a classification of “at least Skin Sens. 1B”. *Positives with unknown DSA* demonstrate the sensitizing properties of the test substance, but do not allow for sub-categorization. Both result types, however, are sufficient to identify the chemical as a sensitizer (Skin Sens. 1, without sub-category).
- *Negative HPPT results at an induction DSA* ≤ 500 µg/cm^2^ suggest that a classification for skin sensitization might not be needed. However, unless the test concentration applied marks the highest achievable concentration (as described above), it cannot be ruled out with sufficient certainty that a positive test result might have been obtained at a higher concentration. The same holds for negative test results for which the test concentration is unknown.
- *Negative HPPT results at an induction DSA* > 500 µg/cm^2^ but below the highest achievable concentration (as described above) suggest no need for classification. However, while classification as Skin Sens. 1A can be ruled out, classification as Skin Sens. 1B cannot, because a higher test concentration might have resulted in a positive test result.

While there is general text in the GHS about using frequency of occurrence and potency level for sub-categorization in humans, another limitation of the current GHS classification scheme for HPPT data is that it does not take into account the number of individuals sensitized in testing, thereby failing to consider a major indicator of potency. If, for example, each of two substances were tested in an HMT with 25 test subjects at an induction DSA of 501 µg/cm^2^, with one producing only one and the other 25 sensitized individuals, this significant difference in potency would be ignored by strict application of the current GHS guidance values, which will classify both substances as Skin Sens. 1B.

In the following analysis we describe how these noted ambiguities may be overcome to some extent by introducing modified classification criteria for skin sensitization based on HPPT data, which, in addition, would better reflect the potency information contained in these data in certain situations.

## Materials and methods

### The HPPT database

As noted above, the work presented here utilized a database of 2277 test results for 1366 unique test substances, which is described in detail in another paper from this group (Strickland et al. 2023). This database contains data generated with two major HPPT designs (the HMT and the HRIPT), both of which are explicitly mentioned in the GHS text. Unless noted otherwise, the evaluations presented in this review are based on this full database.

Importantly, we introduced a “Relative Reliability Score” [RRS, cf. OECD (2021b) and Strickland et al. (2023)] to assess the reliability of individual study results from “highly reliable” (RRS = 1) to “not reliable” (RRS = 5). We only included study results with an RRS of 1–4 (2255 of the total of 2277 results in the database) in the assessment reported here. The reasons for assigning an RRS = 5 are explained in detail in Strickland et al. (2023).

The reference substance list for the OECD DA project initially consisted of the “Cosmetics Europe” reference list of 128 substances, described in detail in Hoffmann et al. (2018). In the course of the OECD work, some of these substances were removed because of variable or ill-defined composition, while other substances were added, mainly to broaden the set of LLNA-negative reference chemicals. The final OECD DA reference list contains 196 reference substances with in vitro data [Direct Peptide Reactivity Assay (DPRA, OECD TG 442C), KeratinoSens (OECD TG 442D), human Cell Line Activation Assay (h-CLAT, OECD TG 442E)] and varying degrees of coverage by LLNA and HPPT data (OECD 2021a, d). In this review, the subset of the full HPPT database associated with these chemicals is referred to as the “OECD DA reference list”.

### Modified classification criteria for single HPPT results

To overcome the limitations of the current GHS criteria regarding the sub-categorization based on single HPPT results, we developed a more sophisticated system of classification to answer the following two questions:

1. If a positive test result (≥ 1 sensitized individual) is obtained at an induction DSA > 500 µg/cm^2^, (how) can the likelihood of a positive outcome at ≤ 500 µg/cm^2^ be determined?
2. Is there a DSA or test concentration < 100% at or above which a negative test result (no sensitized individual) can be accepted as such without confirmation that this DSA or concentration represented the highest achievable value, and (how) can this concentration be determined?

#### DSA1+

To address Question 1 above, we needed to estimate whether the minimum induction DSA causing a positive test result (i.e., the DSA sensitizing exactly one test subject) is less than, equal to or greater than 500 µg/cm^2^. We started by introducing the “*DSA1*+” (i.e., the *hypothetical DSA that sensitizes exactly one test subject*). If test data at different DSAs were available, the number of sensitized individuals could be plotted versus the DSA and a benchmark dose could be derived. However, in practice this is not possible for most HPPT data, which are usually generated using only one test concentration (i.e., only one dose–response data point is available). In this situation, one approach to estimate the DSA1+ is by linear extrapolation of the induction DSA causing the number of positive responses observed in the test to the hypothetical DSA resulting in exactly one positively tested individual, i.e., to calculate the DSA1+ as DSA1+  = DSA/(number of sensitized individuals).

For example, if five individuals were tested positive with an induction DSA of 300 µg/cm^2^ in a given test, the DSA1+ hypothetically resulting in only one sensitized individual would be one fifth of that DSA, i.e., 60 µg/cm^2^. Note that this is not at all meant to imply that the dose–response is actually linear, it is just an approximation in the absence of relevant dose–response information similar to linear inter-/extrapolation used in determining EC3 values from LLNA dose–response information.

The DSA1+ can then be used for classification under the GHS in the same way as the DSA:

- If DSA1+  ≤ 500 µg/cm^2^, then this test result results in classification as Skin Sens. 1A.
- If DSA1+  > 500 µg/cm^2^, then classification as Skin Sens. 1B is appropriate.

**Fig. 1:**
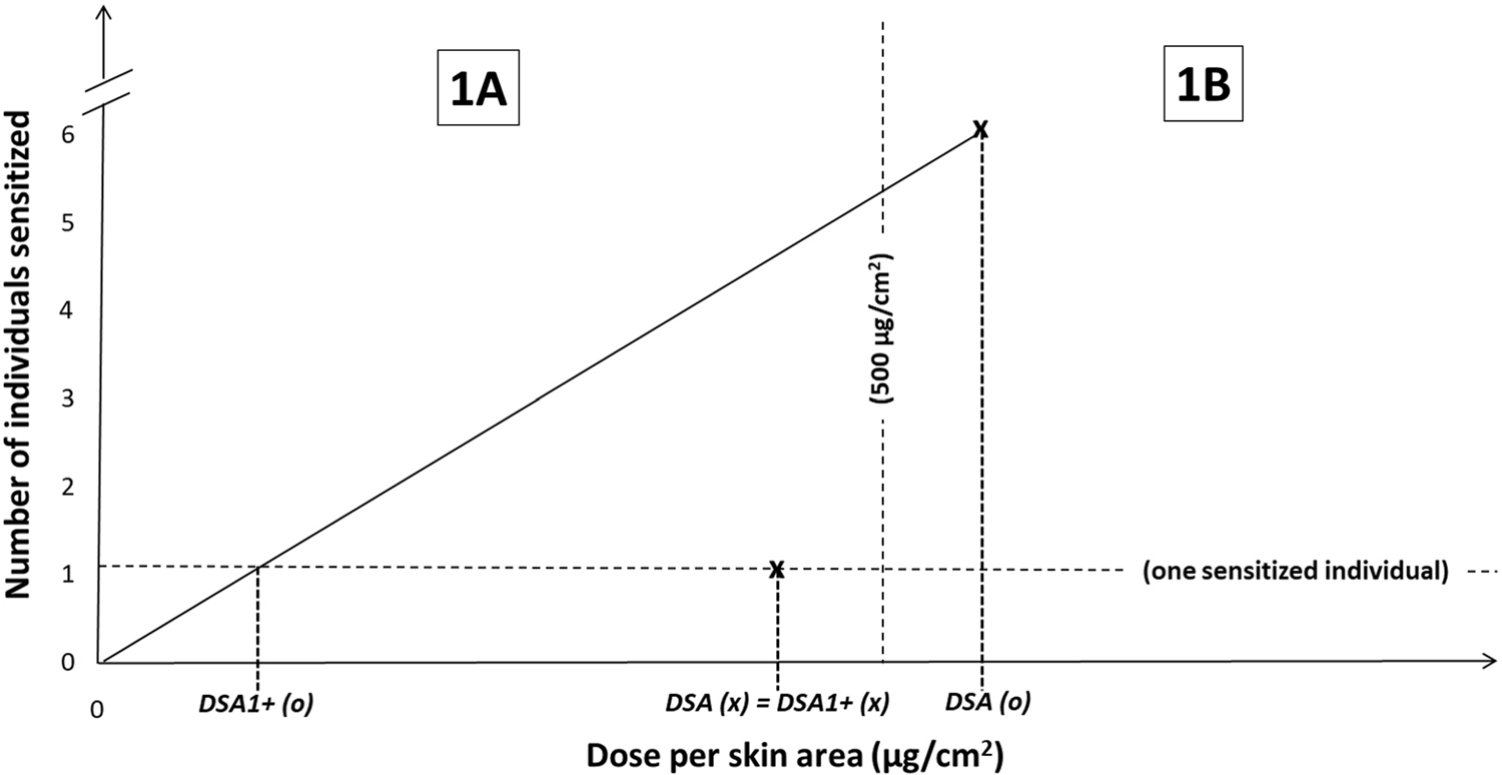
Comparison of classification results based on positive test results for two different substances (x and o), using the current GHS dose descriptor (DSA) and the newly proposed descriptor DSA1+ (see text for details)

As an example, Fig. 1 shows two substances, one (x) causing exactly one sensitized individual, at a DSA (which is also the DSA1+) slightly below the 500 µg/cm^2^ cut-off, and consequentially sub-categorized as Skin Sens. 1A. The other substance (o), tested at a slightly higher DSA above 500 µg/cm^2^, would be sub-categorized as Skin Sens. 1B under the current GHS criteria, regardless of the fact that many more individuals (six) were sensitized. To compare the potency of both substances, the DSA for the second substance is converted to the DSA1+ by extrapolation which now clearly falls into the 1A range.

To differentiate classification outcomes obtained in this way from "standard" GHS classifications, we will call them "extrapolated" classifications.

#### DSA05

As an alternative to the DSA1+, a dose descriptor called "*DSA05*" (i.e., the *induction DSA resulting in 5% of the test panel being sensitized*), has been proposed in the literature (Griem et al. 2003). This parameter is obtained by a linear extrapolation approach in much the same way as the DSA1+ (i.e., DSA05 = [(DSA/% incidence) × 5%].

The current GHS criteria for HPPT data do not use a percent incidence cut-off or threshold (such as 5% sensitized). Instead, classification of the test substance as a skin sensitizer results from the occurrence of one or more sensitized members of the test panel. It is obvious that this may relate to very different sensitization rates depending on panel size [e.g., 1/25 or 4% in the case of the standard HMT designs (Kligman 1966; Kligman and Epstein 1975) and from 1/50 (2%) to 1/200 (0.5%) in the standard HRIPT designs (Draize 1959; Griffith 1969; Jordan and King 1977; Marzulli and Maibach 1973; Marzulli and Maibach 1980; Politano and Api 2008; Shelanski and Shelanski 1953; Voss 1958)].

Thus, the GHS' convention of choosing an absolute incidence threshold limits the comparability of test results obtained with test panels of different sizes. Two substances can be considered equipotent, if under identical test conditions the same dose of both substances leads to the same magnitude and incidence of effect in a given population (Chiu and Slob 2015). Although magnitude of effect is usually not reported with HPPT results, the DSA05 could be a better choice for the direct comparison of relative skin sensitization potencies of two substances than the DSA, as it allows for comparing doses that caused the same incidence of sensitization in humans under comparable test designs, regardless of panel size. In this review, therefore, we compare the classification outcomes based on both, DSA1+ and DSA05.

#### Limit for acceptance of negative results

To address Question 2 above, we also wanted to estimate a minimum concentration or dose, above which it would be unlikely that a test result would be falsely regarded as negative. As the OECD working group analyzed both HPPT and LLNA reference data in parallel, we wanted to conduct both assessments as consistently as possible. Therefore, we chose an approach suitable for both data types, which was based on the test concentration, and not the DSA (normally not available for LLNA test results).

In a first step, we determined for each positive result in the database the *hypothetical concentration leading to exactly one sensitized individual *(*CONC1*+) by linear extrapolation from the observed number of sensitized individuals at the test concentration applied (CONC). In analogy to the approach chosen to calculate the DSA1+ above, CONC1+ is calculated as CONC1+  = CONC/(number of sensitized individuals). We then analyzed the distribution of CONC1+ values for all positive test results in our HPPT database, for which a CONC1+ value could be calculated (n = 592/605 positive test results in total^2^). The median CONC1+ was determined to be 1.3%, the 95th percentile 10% and the 99th percentile 25%.

These numbers suggest that if a negative test result were obtained at a test concentration > 25%, the substance could still be a sensitizer, but its potency would be lower than that of 99% of the substances in the database with a positive result. Based on these findings, we decided to use 25% (the 99th percentile) as the cut-off (i.e., minimum test concentration) for test results to be accepted as negative.

#### Borderline or ambiguous classifications

Given the variability and uncertainty associated with the HPPT data as well as the corresponding ambiguity in classification outcome, we defined a borderline range near the 1A/1B cut-off of 500 µg/cm^2^. For substances with a DSA1+ in this borderline range, there is a higher likelihood of incorrect sub-categorization than for those with DSA1+ values at a greater distance from the cut-off. Since variability and uncertainty around the HPPT data cannot be reliably quantified [cf. OECD (2021b)], the width of the borderline range can only be chosen somewhat arbitrarily. Still, this allows for a more uniform, transparent, and reproducible classification mechanism and is therefore preferable to a subjective, "expert judgment"-based case-by-case approach.

For DSA1+ and DSA05, we chose a borderline range of ± 25% around the 500 µg/cm^2^ cut-off (i.e., from 375 to 625 µg/cm^2^). Negative results with a test concentration < 25% were considered ambiguous (see next section for details).

#### Modified classification approach

Based on the considerations above, we applied the following modified classification approach:

- *For negative test results with CONC* ≥ *25%*, the classification outcome was NC (not classified), regardless of the induction DSA value. This means that according to the criteria of the GHS, this test result does not call for a classification of the test substance as a skin sensitizer; it does not, however, mean that this test result proves that the substance is not a sensitizer.
  *Negative test results with CONC* < *25% obtained at an induction DSA* > *625* µg/cm^2^ (i.e., above the upper boundary of the borderline range around the cut-off between sub-categories 1A and 1B) were assigned the ambiguous classification outcome NC/1B. NC/1B indicates that it is not possible to decide between two GHS classification outcomes (NC or 1B) with sufficient certainty. However, for test results with this outcome, the likelihood that sub-categorization as 1A would be appropriate is considered very low (whereas 1B cannot be excluded).
  In the case of a *negative test result obtained with CONC* < *25% and an induction DSA* ≤ *375* µg/cm^2^ (i.e., below the lower boundary of the borderline range around the 500 µg/cm^2^ cut-off), *or for which CONC was not available*, the ambiguous classification outcome “NC/1” was assigned. This effectively means that the test result cannot provide any decisive information on whether the substance is a skin sensitizer or not. Therefore, such results were excluded from the overall classification process.
- *For positive test results*, the extrapolated classification was 1B, if DSA1+  > 625 µg/cm^2^, and 1A, if DSA1+  ≤ 375 µg/cm^2^
  *Positive test results with 500 µg/cm*^*2*^ < *DSA1*+ ≤ *625* µg/cm^2^ received an extrapolated classification as “1B+”. These test results were interpreted as borderline 1B, showing a moderate sensitization potential (1B), but with some (non-quantifiable) likelihood of underclassification (i.e., of assigning a less strict sub-category than appropriate).
  For some of the positive test results, a DSA1+ value was not available. To such cases, we assigned the classification outcome “1”,^3^ in order to reflect that a reliable GHS sub-categorization (1A or 1B) was not possible.
  *Positive test results with 375 µg/cm*^*2*^ < *DSA1*+ ≤ *500* µg/cm^2^ were classified as “1A−”. These test results were interpreted as borderline to showing a strong sensitization potential (1A), but with some (non-quantifiable) likelihood of overclassification (i.e., assigning a stricter sub-category than necessary).

**Fig. 2:**
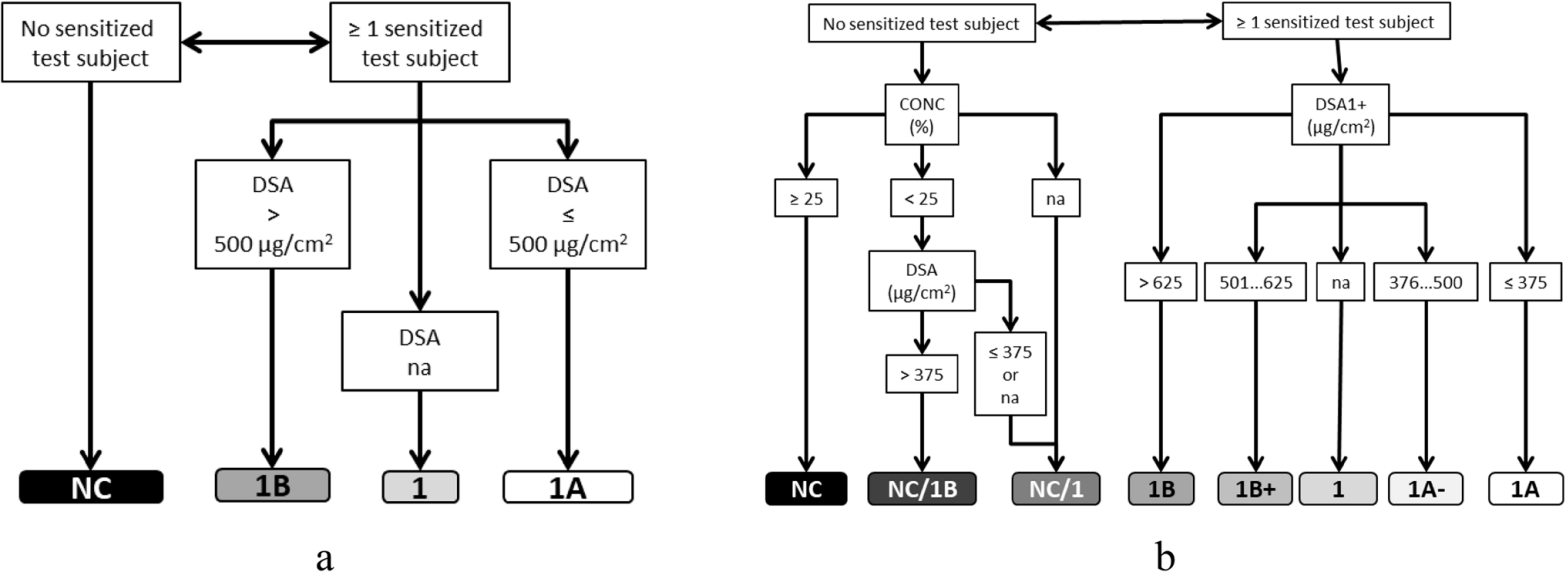
Schematic representation of the logic of the classification process for individual HPPT results according to **a** the current GHS criteria and **b** the modified approach discussed herein for generating “extrapolated classifications”. na = not available

Figure 2 shows schematic representations of the current GHS approach (a) and the modified approach for obtaining extrapolated classifications (b).

### Combination of multiple HPPT results into a WoE classification result

For a considerable number of substances in the HPPT database, multiple HPPT results were available. In cases in which these results were not fully concordant for a given substance (i.e., where they pointed at different classification outcomes), the overall classification outcome had to be determined using a WoE approach. We applied and compared three different WoE approaches for this purpose. Examples of their application are also provided in OECD (2021b).

The three approaches differed from each other with respect to some variation of underlying rules and assumptions. In this way, we were able to perform a *sensitivity analysis* regarding the influence of those variations on the overall classification outcome and, hence, the robustness of the overall WoE conclusion.

#### WoE score method

In this approach, we first determined the extrapolated classification outcomes for the individual test results based on Fig. 2b. Next, each outcome received a numerical score based on the scheme in Table 1.

**Table 1:**
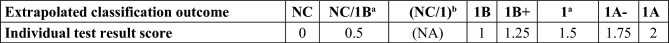
Numerical scores assigned to extrapolated classification outcomes from individual test results

^a^For ambiguous outcomes considered for the overall classification, the average score was used, i.e., NC/1B receives a score of (0 + 1)/2 = 0.5 and 1 a score of (1 + 2)/2 = 1.5

^b^The ambiguous classification outcome “NC/1” was excluded from the overall classification (see text for details)

The scores assigned to each extrapolated classification outcome were chosen intuitively (but also—to a degree—arbitrarily) to reflect a possible way in which a risk assessor could combine multiple HPPT results in a WoE assessment. Test results with the ambiguous outcome NC/1 did not receive a numerical score, nor did we consider them for the overall WoE classification.

We then added up the individual scores from all tests and divided the sum by the number of test results to obtain an overall WoE score for each substance, which was rounded to the second decimal.

#### The “median-like location parameter” (MLLP) method

In addition to the WoE score method, we applied the MLLP approach described for the analysis of LLNA data by Hoffmann and co-workers:

“This parameter was defined as the median for substances with repeat studies with an EC3 in more than 50% of the repeats. For substances with at least 50% negative repeat studies, i.e. no EC3 value was available, the parameter was defined as the modified median. The first step in deriving the modified median was to review the negative studies in detail: when the maximum concentration tested in a given study was lower than the median EC3 of the positive studies for the same chemical, the respective negative study was excluded, because it was considered a limited validity as tested concentrations were too low. From the remaining negative and all positive studies, the median was used as a location parameter (modified median). In the case of 50% of repeat studies being negative and 50% being positive, the highest EC3 value was defined as the modified median.” (Hoffmann et al. 2018).

For adaptation to the extrapolated HPPT-based classification outcomes, however, we needed to further interpret their approach as follows:

- *Test results with the ambiguous classification outcomes NC/1 or NC/1B* were considered negatives in this WoE approach, but only if the database for the chemical under consideration also included studies with a positive outcome (1A, 1B, or 1) and the DSA applied in the NC/1B study was greater than or equal to the median DSA1+ of the positive studies (for which a DSA1+ was available). This rule also implies that if there are only NC/1B outcomes, no MLLP is available.
- *If 50% or more of the study results remaining after the previous step were positive,* the substance was considered a sensitizer, but if they were negative, the overall reference classification was NC.
- For GHS sub-categorization, *test results with a positive, but ambiguous classification outcome 1* were excluded (in addition to the NC/1 and NC/1B studies with test concentrations that were too low, as described above). Then, the MLLP of the remaining study results was calculated. In the case where a substance had an even number of HPPT results factoring into the WoE assessment, and if the median fell between two test results with DSA1+ values, we calculated the MLLP as the average of those two values. If it fell between the highest negative study and the lowest test result with a DSA1+ value, that DSA1+ value was the MLLP.
- If the available individual test outcomes were only NC or NC/1B, the overall MLLP was NC.

#### The “median sensitization potency estimate” (MSPE) method

The MLLP approach as published in Hoffmann et al. (2018) and further interpreted by us has certain weaknesses. In some cases, it produces less strict WoE results than would seem appropriate or intuitive, compared to how different test results might be brought together in a WoE assessment by a regulator tasked with classifying the respective substance for skin sensitization.

To address these weaknesses, we further modified the MLLP approach as follows to obtain a "*Median Sensitization Potency Estimate*" (*MSPE*):

- As for the WoE score method, we began by excluding all *NC/1 test results* from the assessment, since they do not add any relevant information (instead they add noise to the median determination).
- *Positive test results with the outcome 1* (i.e., without an available DSA1+ value) were included when determining the position of the median.
- All test outcomes, whether numerical (DSA1+ values) or categorical (1, NC/1B), were called “Sensitization Potency Estimates” (SPEs, in analogy to the “Acute Toxicity Estimate (ATE) values of the GHS) and the median test result was therefore called the “Median Sensitization Potency Estimate” (MSPE).
- All SPE values were finally arranged in the following order of ascending potency:
  NC → NC/1B^4^ → Numerical SPE (DSA1+) values > 500 µg/cm^2^ in descending order → 1 → Numerical SPE DSA1+ values ≤ 500 µg/cm^2^ in descending order.

The value of the MSPE was then determined as follows:

- *If there were one or more positive results in addition to one or more NC/1B results*, but there was no clear NC result, the median DSA1+ of the positive results with numerical values was taken as the MSPE. However, when the number of 1A (including 1A−) study results equaled that of the 1B (including 1B+) results, the MSPE was 1.
- *If there were one or more NC results and all other test outcomes were NC/1B*, the MSPE was NC.
- In all other cases (i.e., those in which clear positives and negatives were both present and where the median fell between a numerical and a non-numerical result), the numerical result was taken as the MSPE.

#### Determination of the overall classification outcome

The outcome from the individual WoE methods was translated into three different overall classification modes, as shown in Table 2:

- GHS_BIN_: Binary classification scheme [i.e., 1 (sensitizer) or NC (not classified)];
- GHS_SUB_: Sub-categorization scheme including the two GHS skin sensitization sub-categories [i.e., 1A (strong sensitizers) and 1B (other skin sensitizers), as well as NC (not classified)];
- GHS_BORDER_: Same as GHS_SUB_, with the two additional ambiguous classification outcomes (1 and NC/1B). Again, neither 1, nor NC/1B are potency sub-categories; they characterize a limited data situation, where the uncertainty in the assignment of the test substance to a GHS sub-category (or the outcome NC) is high.

**Table 2:**
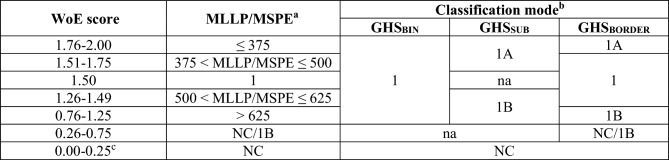
Translation of WoE score, MLLP and MSPE values into overall GHS reference classifications (na = not applicable)

^a^Numerical values are in µg/cm^2^

^b^For GHS_SUB_, individual test results with 1A and 1A− were both considered as 1A, and both 1B and 1B+ were considered as 1B. For GHS_BORDER_, 1A− and 1B+ were considered as 1

^c^In the special case that one or more test results yielded the outcome NC and all other test results had the outcome NC/1B, the WoE score was set to zero

It is noted that for the actual classification/sub-categorization of the OECD DA reference data, we only used GHS_BIN_ and GHS_SUB_ directly. GHS_BORDER_ was only considered as additional information pointing out that some GHS_BIN_ or GHS_SUB_ classification outcomes were associated with higher uncertainty than others. The GHS_BORDER_ information may be important when weighing the outcome from HPPTs against those from other data sources on skin sensitization, such as the LLNA.

*If all three WoE approaches agreed*, the overall outcome was considered robust and was used for further evaluation. The same held if one of the three approaches (WoE score, MLLP or MSPE) did not provide a result, but the outcomes based on the remaining two agreed with each other. If only one of the approaches returned a result, the classification outcome from that approach was used.

*Where the three WoE approaches disagreed regarding the GHS*_*SUB*_* outcome* (i.e., both 1A and 1B outcomes were present), the overall outcome for the respective substance was decided by rule-guided expert judgment on a case-by-case basis. For eight of the 196 OECD DA reference substances, this decision was made following a detailed discussion in the OECD expert group [for details, see Table 17 in OECD (2021b)]. For two (DSA05), or five (DSA1+, including the two substances for DSA05) additional substances in the full database, the decision was made according to the following rules:

1. If the available data for that substance contained one or more positive HPPT result with a DSA ≤ 500 µg/cm^2^ (i.e., a clear 1A outcome according to the current GHS criteria), the outcome was set to 1A.
2. If an overall GHS_SUB_ classification outcome could be obtained using DSA05, but not using DSA1+, the DSA05 outcome was also used for DSA1+.
3. If neither rule 1, nor rule 2 was applicable, the overall outcome was decided on a case-by-case basis.

Discordant GHS_BORDER_ outcomes from the individual WoE approaches (i.e., MLLP, MSPE or WoE score) were first examined to determine whether a *consensus approach* could be applied. The rationale for this was that the ambiguous GHS_BORDER_ outcomes 1 and NC/1B obtained via the individual WoE approaches do not, on their own, allow for a clear decision on the overall classification outcome. However, such results may still support—or at least be compatible (i.e., not in contradiction) with—the outcome of other WoE approaches. For example, the outcome 1 is compatible with the outcomes 1A, 1B, or NC/1B, but incompatible with the outcome NC. Likewise, the ambiguous outcome NC/1B is compatible with the outcomes 1, 1B and NC, but not with the outcome 1A.

As a consequence of these considerations, *if the outcome from one of the available WoE approaches *(*WoE score, MLLP, MSPE*)* was 1A or 1, while another was NC, or if one was 1A and another NC/1B,* the results were considered incompatible, and an expert call was required in analogy to the procedure for GHS_SUB_ above. In all other cases, the *consensus approach* shown in detail in Table 3 was applied.

**Table 3:**
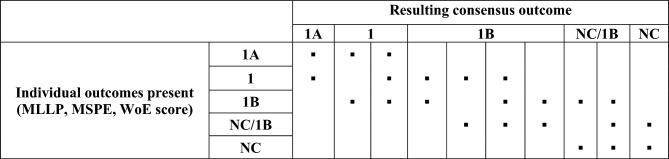
Overview of the decision scheme applied, where possible, to obtain an overall GHS_BORDER_ consensus outcome in case of disagreement between the available individual outcomes (▪ = present, empty = absent) based on WoE score, MLLP, and/or MSPE. In all other cases of disagreement, the overall classification was decided based on expert judgment

It is noted that this rule-based system was developed after publication of OECD (2021b) and therefore some of the overall GHS_BORDER_ outcomes given here may differ from those provided in Table 17 of that publication.

Originally, overall classifications were determined only for the OECD DA reference substances. For the present review, we coded the above rules into a script (supplementary files “HPPT-classification.R” and “HPPT-classification.nb”) using the statistical software R v.4.2.2 (R Core Team 2022). The corresponding overall classifications were then calculated for the full HPPT database of 1366 substances based on 2255 test results with RRS < 5 and can be found in the supplementary file “HPPT-classification.xslx” (tabs “DSA1+” and “DSA05”).

### Reproducibility of HPPT-based WoE classifications

If we could perform an infinite number of HPPTs with a given substance, this would allow for a determination of the “true” HPPT sensitization potency of that substance and, on that basis, its “true” HPPT-based classification or sub-categorization according to the UN GHS. Reproducibility of that classification or sub-categorization could then be measured by a statistical evaluation of all individual HPPT results against the “true” HPPT-based classification. Unfortunately, this is not possible, and reproducibility must instead be estimated from a limited number of test results, using the WoE-based overall classification described above as a surrogate for the “true” classification. Reproducibility can then be understood as the likelihood that the classification outcome derived from an individual HPPT result matches the outcome from the WoE assessment of all results available for the substance in question.

As in OECD (2021b), we therefore determined the reproducibility of the overall classification result in the following way:

- *GHS*_*BIN*_: Reproducibility was calculated as the fraction of all individual HPPT results yielding an unambiguous classification result (1 or NC) for a given chemical that correctly predicted the WoE call based on the three approaches. Studies resulting in an SPE of NC/1 or NC/1B were excluded from this evaluation, since for them GHS_BIN_ was not applicable.
- *GHS*_*SUB*_: Reproducibility was calculated as the fraction of all HPPT results yielding an unambiguous classification result (1A, 1B, or NC) for a given chemical that correctly predicted the WoE call based on the three approaches. Studies resulting in an SPE of 1 or NC/1 were excluded from this evaluation, since for them GHS_SUB_ was not applicable. For the same reason, studies resulting in NC/1B were omitted, if the overall classification was 1B or NC. They were, however, counted as incorrect predictions if it was 1A.

### WoE assessment of HPPT- and LLNA-based reference classifications

In the OECD DA project, LLNA-based reference classifications were established using an approach analogous to the one described here for the HPPT data (OECD 2021c). Although outside the scope of the OECD DA project, we compared both classifications, where available for the same substance. It is important to note that we only performed a WoE assessment of the HPPT and LLNA data available to us in the frame of the OECD project. A true overall WoE assessment for the substances in question would need to consider all available data relevant for skin sensitization classification including non-HPPT human data, data from tests in guinea pigs and data obtained from in vitro tests, DAs or in silico models.

HPPT- and LLNA-based results were considered *concordant*, if they were identical or at least not in contradiction to each other:

- The outcome NC/1B was considered concordant with the outcomes 1, 1B and NC.
- The outcome 1 was considered concordant with the outcomes 1A and 1B.

**Fig. 3:**
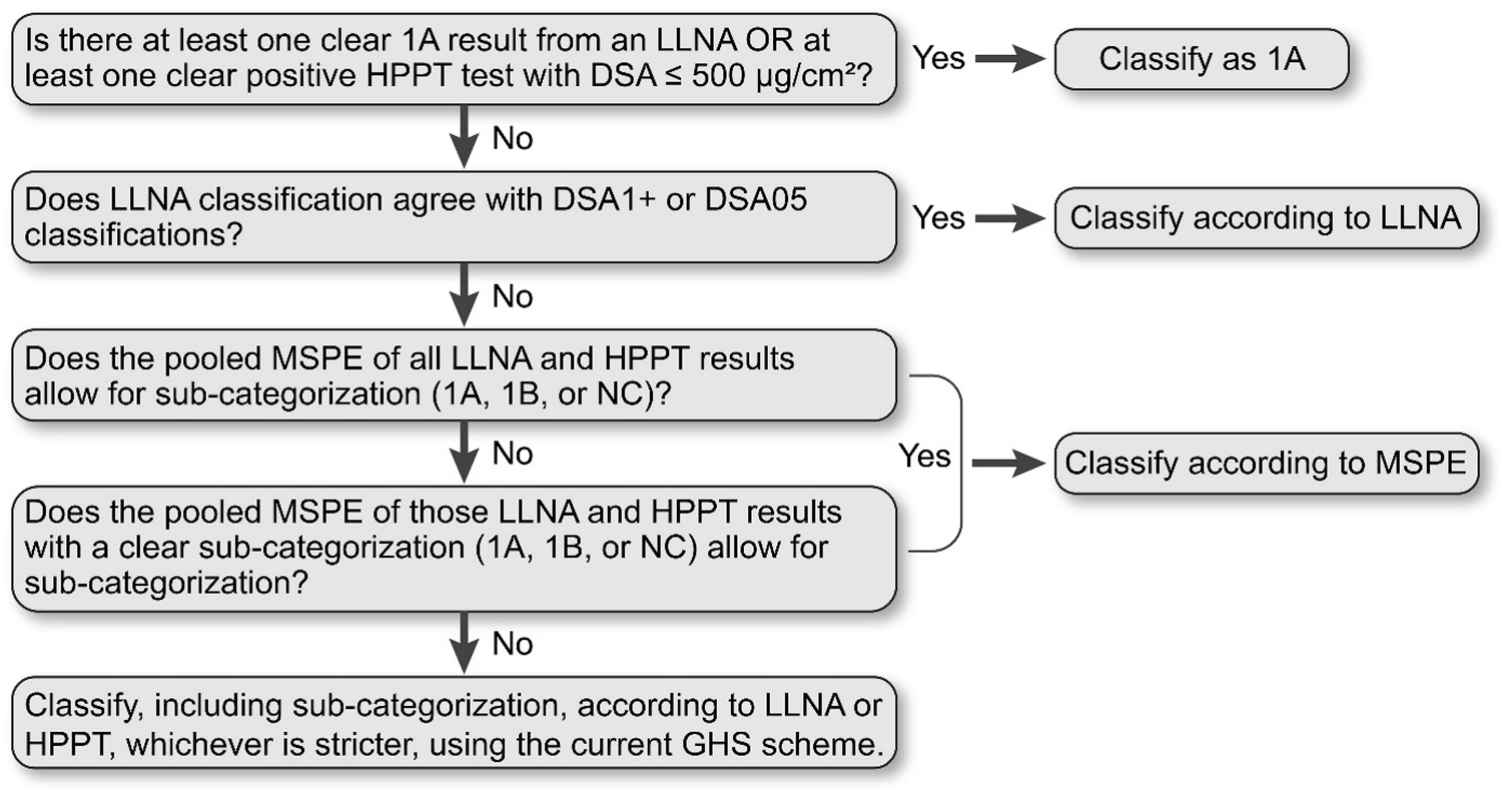
Decision scheme for obtaining an overall classification based on all available LLNA and HPPT data, in cases where the three individual classifications based on LLNA, DSA1+ and DSA05 did not fully agree

Discordant results were resolved by applying the decision logic shown in Fig. 3, which, again, aims to represent regulatory practice as closely as possible.

In short, the basis for this stepwise scheme was as follows:

- *The presence of one or more clear 1A results* in the LLNA database led to classification of the substance as 1A.
- *A positive HPPT result with a DSA* ≤ *500 µg/cm*^*2*^ in the absence of a clear LLNA 1A result led to classification of the substance as 1A.
- If these rules did not apply, and there was *disagreement between DSA1*+ *and DSA05*, classification was decided according to the LLNA result. This can also be seen as a majority vote, since in these cases the LLNA agreed with either DSA1+ or DSA05.
- For substances not classifiable by any of the three preceding steps, all LLNA and HPPT results (except for those resulting in the outcome NC/1) were combined to determine the overall MSPE according to the rules given in the “The “median sensitization potency estimate” (MSPE) method” section. This was done in parallel using DSA1+ and DSA05 values, with the resulting sub-categorization accepted only if the two MSPE results obtained in this way agreed with each other.
- If sub-categorization was still not possible, determination of the MSPE was repeated in parallel based on DSA1+ and DSA05 as described above, using only test results with unambiguous outcomes (1A, 1B, NC).
- Finally, if still no sub-categorization was obtained, the LLNA-based classification was compared to the HPPT-based classification determined using the current GHS scheme rather than the extrapolated classification outcomes introduced in this manuscript. The stricter of the two classifications (including subclassification) was then applied as the overall WoE outcome. For example, if the LLNA-based classification was Skin Sens. 1B and the HPPT-based classification was Skin Sens. 1A using DSA1+ or DSA05, but Skin Sens. 1B using the DSA according to the current GHS classification scheme, the overall WoE classification was 1B.

It is noted that the above rules pertain to the WoE-based determination of GHS_BIN_ and GHS_SUB_. In all cases, GHS_BORDER_ was chosen to reflect the disagreement between LLNA, DSA1+ and/or DSA05.

## Results

### Individual test results

#### Classification of the full HPPT database

Individual study results available for the OECD DA reference list chemicals have been reported in OECD (2021b). Individual study outcomes for the full database are available on the website of the U.S. National Toxicology Program (NTP).^5^

The full HPPT database comprises 2277 test results, 2255 of which were found sufficiently reliable for inclusion in the further analyses. Of these 2255 test results, 605 were positive (≥ 1 sensitized test subject) and 1650 negative (0 sensitized subjects).

**Fig. 4:**
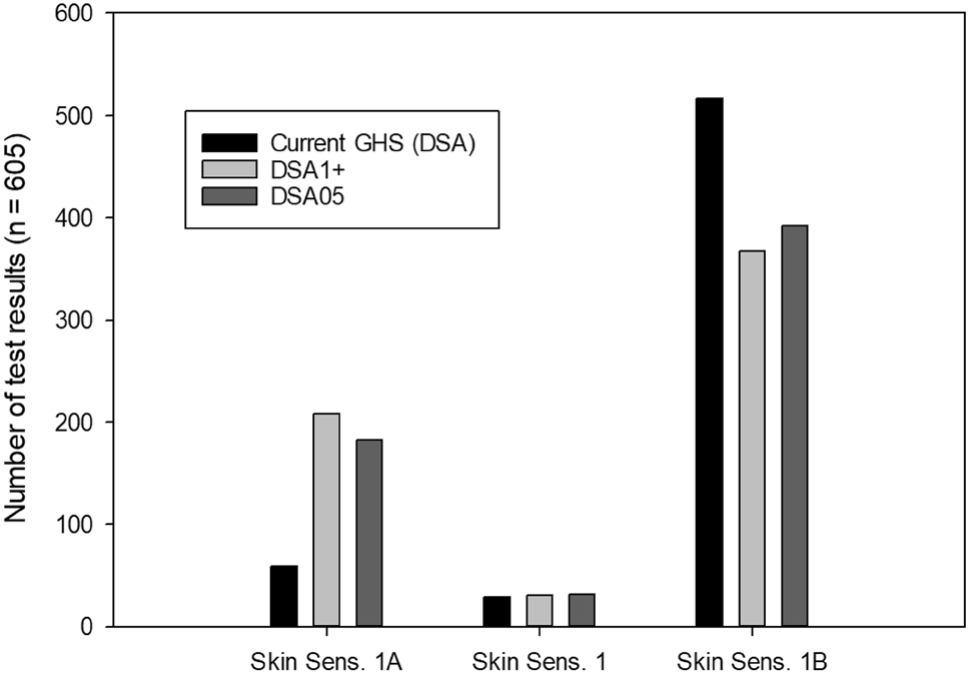
Overview of the classification outcomes from the individual test results in the HPPT database (n = 2255) when applying the current GHS approach (Fig. 2a) or the modified approach (Fig. 2b) using DSA1+ or DSA05 as dose descriptors

Figure 4 shows the classification results obtained from positive test results by using the current GHS approach, the DSA1+, and the DSA05, respectively.

Note that these numbers represent individual test results, not substances, and therefore multiple test results may belong to the same substance. Considering all test results, the current GHS approach resulted in a clearly less strict sub-categorization outcome (i.e., a smaller number of 1A and a higher number of 1B outcomes) compared to the two other approaches.

Of the 1650 negative test results, only 69 (4.2%) were assigned the unambiguous outcome of NC (i.e., accepted as negatives) based on either a test concentration of 25% or higher or on expert judgment confirming that the maximum achievable test concentration had been applied. If a slightly higher likelihood of error were allowed by using the 97th percentile (a test concentration of 20%) of the CONC1+ distribution as the lower limit for accepting a negative test result as such, then the number of unambiguous negative test results would increase to 183/1650, or 11.1% of the negative test results.

Of the 1650 negative test results, 1416 or 85.8% were ambiguous (classification outcome NC/1B) because the test concentration applied was too low to exclude a 1B sub-categorization outcome, which might have been possible if the negative test result was observed at a higher test concentration. However, the concentration tested was high enough to exclude the possibility of the substance being a 1A sensitizer.

For 165 of the 1650 negative test results (10.0%), the experiments were carried out at such low test concentrations that not even an outcome of 1A could be excluded if testing had been performed at higher concentrations. These results contained no relevant information on skin sensitization potential and were therefore not considered further.

Table 4 shows the results of a pairwise comparison of the classification outcomes from individual HPPTs obtained using DSA1+ or DSA05 as dose descriptors.

**Table 4:**
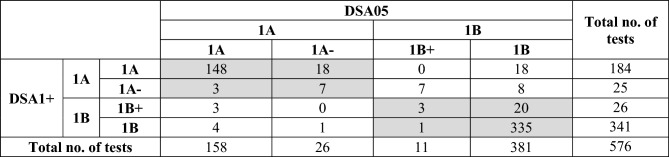
Comparison of the impact of using DSA1+ or DSA05 on potency sub-categorization (n = 576)

Test results for which DSA1+ and DSA05 resulted in the same potency sub-category are shown in shaded gray

In the great majority of cases (535/576 or 92.9%, cells shaded gray in Table 4), both dose descriptors resulted in the same potency sub-category. Where the results disagreed, DSA1+ led to a stricter sub-categorization than DSA05 in a slightly higher number of cases (33/576 or 5.7%) than the other way around (8/576 or 1.4%).

#### Limit for acceptance of negative test results

As explained in the “Limit for acceptance of negative results” section above, we used the 99th percentile CONC1+ value of 25% to define the test concentration at which a negative test result could be accepted for the OECD project. Of note, if 5% is taken as the critical incidence to define a positive test result rather than one sensitized individual, (i.e., DSA05 rather than DSA1+ is used as the dose descriptor for classification), the acceptable minimum test concentration would have to be derived based on the distribution of CONC05, rather than CONC1+ values. For CONC05, the median, 95th and 99th percentile concentration values were 2%, 31%, and 99%, respectively.

### Classification outcomes for individual substances

#### Classification of the full HPPT database

Detailed information on the WoE-based classification results for the OECD DA reference substances has already been reported in OECD (2021b). Here we report the results for the full database of 1366 individual substances for which at least one HPPT result with RRS < 5 was available, as obtained by using the R script referenced in the “Determination of the overall classification outcome” section. The results are shown in Table 5 in the form of confusion matrices using both dose metrics, DSA1+ and DSA05.

**Table 5:**
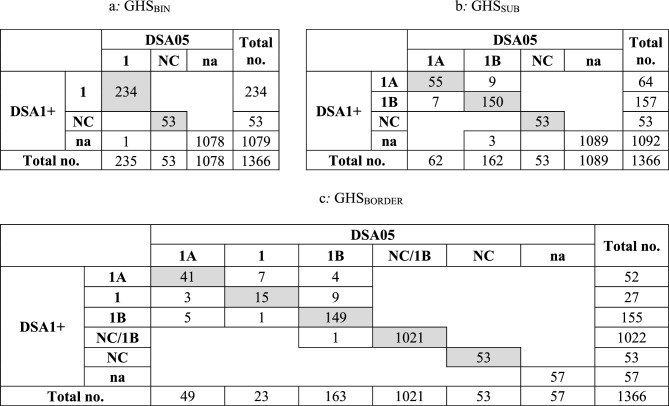
Confusion matrices of the GHS_BIN_, GHS_SUB_ and GHS_BORDER_ classifications obtained for 1366 unique substances for which at least one HPPT result with an RRS < 5, using DSA1+ or DSA05 as the relevant dose metric (na = not available; fields for which both DSA1+ and DSA05 provided identical classification outcomes are shown in shaded gray)

Table 5 shows that the overall concordance of the DSA1+—and DSA05-based approaches is very high when comparing the outcomes for those cases where both were available (shaded gray in Table 5): concordant results were obtained for 287/287 (100%) of the GHS_BIN_ (Table 5a), 258/274 (94.2%) of GHS_SUB_ (Table 5b) and 1279/1309 (97.7%) of the GHS_BORDER_ (Table 5c) classification outcomes.

However, an unambiguous GHS_BIN_ classification as either 1 or NC was obtained based on both DSA1+ and DSA05 for only a minority (287/1366 or 21.0%) of the substances (Table 5a).

For GHS_SUB_, the number of substances with unambiguous sub-categorization as 1A or 1B or the outcome NC was only slightly smaller (274/1366 or 20.0% of the substances, Table 5b).

For GHS_BORDER_, as illustrated in Table 5c, the vast majority of substances (i.e., 1021 (74.7%) or 1022 (74.8%) of the 1366 substances) received the ambiguous classification of NC/1B using DSA1+ or DSA05. As explained in the “Current GHS classification criteria for HPPT data” section, this is a direct consequence of the fact that the majority of the test results in the database was negative (i.e., no sensitization was observed in the test panel) but obtained at such low test concentrations/DSA values that a positive result at a higher concentration/DSA could not be ruled out with sufficient certainty. For an additional 57 substances, a GHS_BORDER_ classification could not be determined because the HPPT studies available for these substances exclusively resulted in the outcome NC/1.

As already evident from Fig. 4, using DSA05 resulted in a slightly smaller fraction of substances sub-categorized as 1A than using DSA1+: 9/64 (14%) substances classified as 1A by DSA1+ were classified as 1B when using DSA05, while 7/62 (11.3%) substances classified as 1A by DSA05 were classified as 1B when using DSA1+.

#### Agreement of different WoE approaches

As shown in the “Individual test results” section, an unambiguous classification outcome could not be obtained for many substances in the database. However, we compared the outcome from the three different WoE approaches (WoE score, MLLP or MSPE) where this was possible. Table 6 shows that where outcomes from two or three WoE approaches (WoE score, MLLP, MSPE) were available, they were highly concordant. For GHS_BIN,_ they were 100% in agreement, while for GHS_SUB_, the outcomes from the three approaches agreed for 250 (97.3%, DSA1+) or 261 (98.5%, DSA05) substances, with the classification of the remaining few substances (DSA1+: 7 or 2.7%, DSA05: 4 or 1.5%) resolved by rule-guided expert judgment.

**Table 6:**
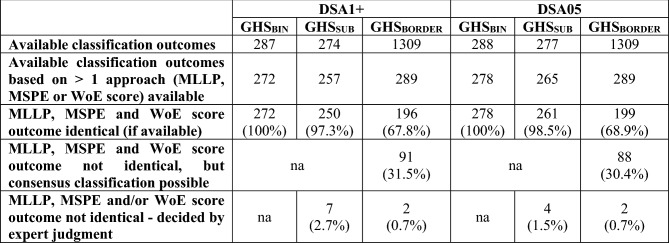
Concordance of the different WoE approaches (MLLP, MSPE, WoE score) with respect to the obtained classification outcomes (in parentheses: percentage in relation to the full number of substances for which the outcome from more than one WoE approach was available)

Notably, for GHS_BORDER_, classification outcomes for most (1020 or 78.0%) of the 1309 substances relied on the result from one WoE approach only (WoE score in all of these cases). Of the remaining 289 substances, however, approximately two-thirds had a concordant classification (196 for DSA1+ and 199 for DSA05). For the remaining third (91 substances for DSA1+ or 88 substances for DSA05), a consensus classification outcome was obtained in all but two cases, where the outcome from the MSPE was 1A, while the outcome from the WoE score was 1B, with the MLLP pointing to NC/1B. As both substances also had a GHS_BIN_ classification outcome of 1 (i.e., GHS_SUB_ not available), we decided to also set the GHS_BORDER_ classification to 1.

### Reproducibility of HPPT-based WoE classification outcomes based on DSA1+

The evaluation in the previous section included all 1309 substances for which at least one test result with RRS < 5 and an outcome other than NC/1 was available. Of these, only 300 had more than one test result. Table 7 summarizes the results of the reproducibility calculations for the HPPT-based overall classifications for substances with at least two test results relevant to the respective classification mode (GHS_BIN_/GHS_SUB_). The detailed data are provided in the supplementary file “HPPT-classification.xslx” (tabs “DSA1+” and “DSA05”, columns J and K).

**Table 7:**
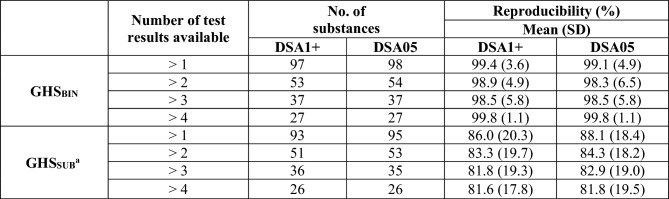
Reproducibility of the HPPT-based overall classifications

^a^Substances for which GHS_SUB_ was decided on by expert judgment are not included

The mean reproducibility of the GHS_BIN_ classification was 98% or greater (98.3–99.8%), indicating that very few of the available test results disagreed with the overall classification outcome. For GHS_SUB_, the mean reproducibility was 82% or greater (81.6–88.1%), with slightly higher values for DSA05-based results.

### WoE with LLNA-based reference classifications

We also investigated the integration of the HPPT-based reference classifications with those obtained using LLNA data. For GHS_BIN_, 56 of the 196 OECD substances had reference classifications based on both data types (47 for GHS_SUB_). Table 8 shows the results of the pairwise comparison of DSA1+ and DSA05 classification outcomes for the OECD DA reference substances with those based on LLNA data.

**Table 8:**
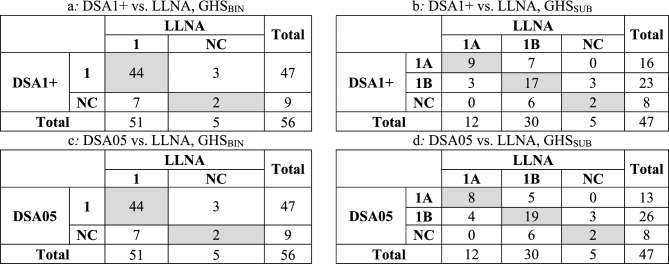
Concordance of HPPT-based classifications with those based on LLNA data for the GHS_BIN_ and GHS_SUB_ classification modes (shown in shaded gray)

Overall concordance (i.e., the percentage of substances for which both HPPT and LLNA data gave a concordant classification) for GHS_BIN_ was 82.1% (46/56 substances, Table 8a and Table 8c, no difference between DSA1+ and DSA05). However, the balanced accuracy of the HPPT-based results for predicting LLNA outcomes, which takes into account the true positive rate (44/47 or 93.6%) and the true negative rate (2/9 or 22.2%), was only 57.9%.

For GHS_SUB_, overall concordance was 59.6% (28/47 substances) for DSA1+ and 61.7% (29/47 substances) for DSA05 (Table 8b and Table 8d). When compared to the LLNA, the HPPT-based approaches were underpredictive (i.e., prediction of 1B instead of 1A or NC instead of 1B) for 9 substances when using the DSA1+ and 10 substances when using DSA05. They were overpredictive (i.e., prediction of 1A instead of 1B or 1B instead of NC) for 10 (DSA1+) or 8 (DSA05) substances, respectively. There was no over- or underclassification of the HPPT-based approaches versus LLNA with respect to 1A and NC (i.e., 1A instead of NC or vice versa).

It should be highlighted that no decision can be made regarding which of the respective classifications (i.e., determined based on DSA1+, DSA05 or LLNA) is the correct or “true” one. On the one hand, the GHS shows a clear preference for data generated in humans, but the HPPTs are characterized by high variability and uncertainty. The LLNA, on the other hand, offers a highly standardized and established test design which has for a long time been a widely used standard assay for regulatory purposes.

For 10 (GHS_BIN_) and 19 or 18 (GHS_SUB_) substances which were not fully concordant (agreement of all three classifications, i.e., from LLNA, DSA1+ and DSA05), the decision tree in Table 3 was applied. In this way, a WoE-based classification could be determined for all 56 (GHS_BIN_) or 47 (GHS_SUB_) substances (for details, refer to the supplementary file “HPPT-classification.xlsx”, tab “LLNA_VS_DSA”). There was no clear trend with respect to whether HPPT or LLNA data were more decisive for the overall WoE classification outcome. In 5 of the 10 cases in which HPPT- and LLNA based GHS_BIN_ classifications were not in concordance, the WoE classification was determined by the HPPT classification, while the other 5 were determined by the LLNA classifications. Of the 19 or 18 classifications with non-concordant GHS_SUB_ classifications based on DSA1+ or DSA05, the WoE classification was determined by the LLNA classification in 11 cases and by the HPPT classification in eight (DSA1+) or 7 (DSA05) cases. Table 9 shows the predictivity of LLNA, DSA1+ and DSA05 for the overall WoE classification for the 56 substances (GHS_BIN_) and the 47 substances (GHS_SUB_) for which all three inputs (i.e., LLNA, DSA1+ and DSA05) were available. The overall concordance values for the LLNA were 91.1% (GHS_BIN_) and 83.0% (GHS_SUB_); for the DSA1+ they were 91.1% (GHS_BIN_) and 76.6% (GHS_SUB_), and for the DSA05 they were 91.1% (GHS_BIN_) and 74.5% (GHS_SUB_).

**Table 9:**
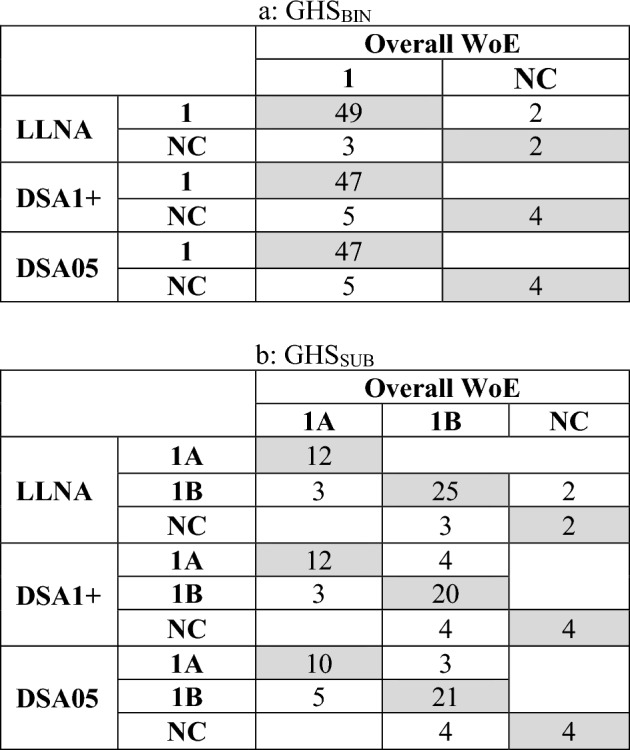
Overview of the integration of LLNA and HPPT (DSA1+ and DSA05) results for the 56 OECD DA reference substances into an overall WoE assessment (cells shown in shaded gray: concordance of the individual approaches with the overall WoE classification outcome)

## Discussion

In this work, based on a comprehensive and well-curated database of 2277 HPPT results for 1366 unique test substances, we were able to gain new insights into the potential but also the weaknesses of using HPPT data the classification of skin sensitizers under the GHS. In this context we have presented:

- a concept for better reflecting potency in the evaluation of individual HPPT results for classification/sub-categorization purposes,
- a WoE assessment approach integrating multiple, discordant HPPT results and allowing for a (limited) sensitivity analysis as well as for the establishment of borderline cases, and
- a simple set of criteria for integrating HPPT results with discordant LLNA data, which could in principle also be applied to other data types suitable for classification of skin sensitizers under the GHS.

HPPT data are often associated with considerable uncertainty and ambiguity. In many cases, the available HPPT data, in particular those with no sensitization observed, do not allow an unambiguous classification on their own. The GHS mentions positive HPPT results as suitable human evidence to support classification, including sub-categorization. And indeed we found that where an unambiguous classification outcome could be established, which was the case for about 20% of the substances in our database, these classifications proved to be highly reproducible.

The current GHS HPPT scheme insufficiently considers, and in some cases may underrepresent, the potency of skin-sensitizing chemicals. To improve the system to better reflect potency, we propose that the DSA1+ and/or DSA05 are used as the relevant dose descriptors rather than the DSA. Both deliver highly concordant results, with the DSA05 on average leading to slightly less conservative classification outcomes compared to the DSA1+. While the induction DSA1+ better reflects classification based on a positive HPPT response as defined by at least one sensitized test subject, the induction DSA05 has the advantage to refer to a benchmark response (5% incidence) that is better comparable across test results obtained with different test panel sizes.

As both dose descriptors have their advantages and drawbacks, perhaps the best choice for the actual assessment of the skin-sensitizing potential of a chemical is to calculate both parameters and make a case-by-case decision when they produce discordant outcomes. It is also noted that the concept of “extrapolated” classification presented here has inherent uncertainties on its own and therefore it is advisable to always consider the non-extrapolated DSA. In cases of doubt, then the ultimate decision on classification could still be made based on the DSA.

Furthermore, we have extensively discussed how to combine multiple HPPT test results into an overall WoE assessment. We recommend application of all three methods used here (WoE score, MLLP and MSPE) as a sensitivity test. In the great majority of cases, all three parameters, where available, produced highly concordant classification outcomes, both with respect to hazard characterization and potency sub-categorization. Few discordant results were observed, which in all cases could be resolved by rule-guided expert decisions [for examples, refer to OECD (2021b)].

Classifications based on multiple HPPT results are generally highly reproducible, both for hazard classification and sub-categorization. Where inconsistencies are observed, generally the test data have quality issues or the respective substances are borderline cases, with a potency near the cut-off between GHS 1A and 1B, or sensitization occurring only at very high test concentrations/DSA values. Some examples from the OECD DA reference data set are discussed in more detail in OECD (2021b).

Finally, we were able to show that classification outcomes based on HPPT data can be successfully combined with other in vivo skin sensitization data, such as from the LLNA, in an overall WoE assessment. In fact, for the substances from the OECD DA reference dataset, HPPT data often complemented the LLNA data set by providing classification outcomes where no LLNA was available. Where HPPT and LLNA data disagreed, a consensus/WoE classification outcome could be established in all cases, following a simple decision logic mimicking the considerations a risk assessor might apply when performing a WoE assessment of such data. Notably, in some of these cases, the overall WoE outcome was determined by the HPPT-based classification, while in others, the LLNA-based classification was considered more robust and therefore taken as the overall classification outcome.

Our work has demonstrated that there is potential for improving the current criteria for using HPPT data for classification under the GHS, in particular with a view to better reflecting potency. As a follow-up activity to this work, we therefore suggest that the findings presented here are further discussed at the UN level with a view to updating the respective GHS text on HPPT-based classification.

### Supplementary Information

Below is the link to the electronic supplementary material.

Supplementary file 1 (R 31 KB)

Supplementary file 2 (HTML 1163 KB)

Supplementary file 3 (XLSX 525 KB)

## Data Availability

All data relevant to this publication are freely available as part of the supplementary material or in the database described in Strickland et al. (2023).

## Footnotes

1 Of note, the GHS describes the classification of substances and mixtures. However, the work presented here focuses on the classification of substances, while further work would be needed to transfer its results to mixtures.

2 For 13 test substances, a CONC1 + value could not be calculated since the exact number of sensitized individuals was not known.

3 Note that in (OECD 2021a, b, c, d), this outcome, when referring to individual test results, was denoted as “POS”.

4 Only those with a sufficiently high test concentration were included, cf. subsection on MLLP method.

5 https://ntp.niehs.nih.gov/go/hppt, last accessed 2023–11-25.

## References

[CR1] Chiu WA, Slob W (2015). A unified probabilistic framework for dose–response assessment of human health effects. Environ Health Perspect.

[CR2] Draize JH (1959) Dermal toxicity. In: Appraisal of the safety of chemicals in foods, drugs and cosmetics, Chapter 6, pp 46–59. The Association of Food & Drug Officials of the United States, Austin, Texas, USA. https://babel.hathitrust.org/cgi/pt?id=uc1.b3596550;view=1up;seq=5. Last accessed 2023 Nov 25

[CR3] Griem P, Goebel C, Scheffler H (2003). Proposal for a risk assessment methodology for skin sensitization based on sensitization potency data. Regul Toxicol Pharmacol.

[CR4] Griffith JF (1969). Predictive and diagnostic testing for contact sensitization. Toxicol Appl Pharmacol.

[CR5] Hoffmann S, Kleinstreuer N, Alépée N, Allen D, Api AM, Ashikaga T, Clouet E, Cluzel M, Desprez B, Gellatly N, Goebel C, Kern PS, Klaric M, Kühnl J, Lalko JF, Martinozzi-Teissier S, Mewes K, Miyazawa M, Parakhia R, van Vliet E, Zang Q, Petersohn D (2018). Non-animal methods to predict skin sensitization (I): the Cosmetics Europe database. Crit Rev Toxicol.

[CR6] Jordan WP, King SE (1977). The development of allergic contact dermatitis in females during the comparison of two predictive patch tests. Contact Dermatitis.

[CR7] Kligman AM (1966). The identification of contact allergens by human assay: II. Factors influencing the induction and measurement of allergic contact dermatitis. J Investig Dermatol.

[CR8] Kligman AM, Epstein W (1975). Updating the maximization test for identifying contact allergens. Contact Dermatitis.

[CR9] Marzulli FN, Maibach HI (1973). Antimicrobials: experimental contact sensitization in man. J Soc Cosmet Chem.

[CR10] Marzulli FN, Maibach HI (1980). Contact allergy: predictive testing of fragrance ingredients in humans by Draize and Maximization methods. J Environ Pathol Toxicol.

[CR11] OECD (2021a) Guideline no. 497: defined approaches on skin sensitisation. 10.1787/b92879a4-en

[CR12] OECD (2021b) Annex 4: Report of the human data sub-group on the curation and evaluation of the human reference data and the derivation of associated substance classfications. In: Supporting document to the OECD guideline no. 497 on defined approaches for skin sensitisation. Series on testing and assessment no. 336. Organisation for Economic Co-operation and Development, Paris. https://one.oecd.org/document/ENV/CBC/MONO(2021)11/ann4/en/pdf. Last accessed 2023 Nov 25

[CR13] OECD (2021c) Annex 3: report of the sub-group on the curation and evaluation of the local lymph node assay reference data and the derivation of associated substance classfications according to the UN GHS. In: Supporting document to the OECD guideline no. 497 on defined approaches for skin sensitisation. Series on Testing and Assessment No. 336. Organisation for Economic Co-operation and Development, Paris. https://one.oecd.org/document/ENV/CBC/MONO(2021)11/ann3/en/pdf. Last accessed 2023 Nov 25

[CR14] OECD (2021d) Supporting document to the OECD guideline no. 497 on defined approaches for skin sensitisation. Series on Testing and Assessment No. 336. ENV/CBC/MONO(2021)11. Organisation for Economic Co-operation and Development, Paris. https://one.oecd.org/document/ENV/CBC/MONO(2021)11/En/pdf. Last accessed 2023 Nov 25

[CR15] Politano VT, Api AM (2008). The Research Institute for Fragrance Materials' human repeated insult patch test protocol. Regul Toxicol Pharmacol.

[CR16] R Core Team (2022) R: a language and environment for statistical computing v. 4.2.2. R Foundation for Statistical Computing, Vienna, Austria. https://www.R-project.org/

[CR17] Shelanski HA, Shelanski MV (1953). A new technique of human patch tests. Proc Sci Sect Toilet Goods Assoc.

[CR18] Strickland J, Abedini J, Allen DG, Gordon J, Hull V, Kleinstreuer N, Ko H-S, Matheson J, Thierse H-J, Truax J, Vanselow JT, Herzler M (2023). A database of human predictive patch test data for skin sensitization. Arch Toxicol.

[CR19] UN (2023) Globally harmonized system of classification and labelling of chemicals (GHS). ST/SG/AC.10/30/Rev.10. United Nations, New York and Geneva. https://unece.org/sites/default/files/2023-07/GHS%20Rev10e.pdf. Last accessed 2023 Nov 25

[CR20] Voss JG (1958). Skin sensitization by mercaptans of low molecular weight. J Investig Dermatol.

