# Supplementary material for: Use of human predictive patch test (HPPT) data for the classification of skin sensitization hazard and potency
Source: Arch Toxicol. 2024 Mar 14;98(5):1253–69. doi: 10.1007/s00204-023-03656-4 (PMC10965744; doi:10.1007/s00204-023-03656-4)
Supplement: Supplementary file 2 — (HTML 1163 KB) [file 204_2023_3656_MOESM2_ESM.html]

Using HPPT Data to Classify Chemicals with Respect to Their Skin Sensitisation Potential


Code 

- Show All Code
- Hide All Code
- Download Rmd

# Using HPPT Data to Classify Chemicals with Respect to Their Skin Sensitisation Potential

#### Kim To

#### 22 November, 2023


The Organization for Economic Co-operation and Development (OECD)
Expert Group on Defined Approaches for Skin Sensitization (EG DASS)
curated a Human Predictive Patch Test (HPPT) database as a reference set
for evaluating DASS [1]. The human data sub-group (HDSG) of the OECD EG
DASS evaluated the HPPT data for classification of chemicals as skin
sensitizers under the United Nations’ Globally Harmonized System of
Classification and Labelling of Chemicals (GHS) [2]. The HDSG developed
a modified classification approach based on two extrapolated response
values:

- DSA1+ - the hypothetical dose-per-skin-area that sensitizes exactly
  one test subject
- DSA5% - the hypothetical dose-per-skin-area that sensitizes 5% of
  test subjects

A weight-of-evidence approach was then developed to combine multiple
HPPT results for a chemical into an overall GHS classification.

This R notebook demonstrates the application of the modified
classification and weight-of-evidence approaches to the HPPT
database.

---

[1] OECD. Guideline No. 497: Defined Approaches on Skin
Sensitisation. 2021. https://doi.org/10.1787/b92879a4-en

[2] OECD. Annex 4: Report of the Human Data Sub-Group on the Curation
and Evaluation of the Human Reference Data and the Derivation of
Associated Substance Classfications. 2021. Series on Testing and
Assessment No. 336. https://www.oecd.org/chemicalsafety/testing/series-testing-assessment-publications-number.htm

# 1 Source Code

The source code for deriving the classifications is saved as an
external R file. Contents of the file are shown here. Sourcing the file
will load the dplyr, readr, and tidyr packages. In addition, this
notebook calls functions from the readxl and openxlsx packages.

```
source("HPPT-classification.R")

# Print contents of file
readLines("HPPT-classification.R") |> cat(sep = "\n")
```


```
#=============================================================================#
# File Name: HPPT-classification-functions.R
# Original Creator: ktto
# Date Created: 2022 Sept 19
# Description: Source code to derive HPPT classifications
# Required Packages:
# - dplyr, readr, tidyr
#=============================================================================#

if (!require(dplyr)) install.packages(dplyr); library(dplyr)
if (!require(readr)) install.packages(readr); library(readr)
if (!require(tidyr)) install.packages(tidyr); library(tidyr)

# GENERAL FUNCTIONS -----

#=================#
# Custom rounding method so rounding behaves as expected
#-----------------#
# `x` = numeric value or vector
# `digits` = number of digits to round to
#-----------------#
# Example: Compare round(1.125,2) and rnd(1.125, 2)
#=================#
rnd <- function(x, digits) {
  dscale <- 10^digits
  z <- trunc((abs(x) * dscale) + 0.5 + sqrt(.Machine$double.eps))
  (z * sign(x)) / dscale
}

#=================#
# Given a sorted vector, returns the value(s) at the middle position of the
# vector
#-----------------#
# sorted_values : a vector of values, assumed to be sorted
#-----------------#
# If provided a vector with an odd number of values, returns the single
# value in the middle position of the vector. If provided a vector with
# an even number of values, returns the two values in the middle position
# of the vector
#=================#
get_median <- function(sorted_values) {
  n <- length(sorted_values)
  if (n %% 2 == 1) {
    sorted_values[ceiling(n / 2)]
  } else if (n %% 2 == 0) {
    i <- n / 2
    j <- i + 1
    sorted_values[i:j]
  }
}

#=================#
# Converts columns to numeric. If the values are given as a range
# or mixture, the minimum value will be returned.
#-----------------#
# `dt` = data frame
# `col_nm` = name of column in dt to be converted (string)
# `na.values` = values to be interpreted as NA
# `match_pattern` = pattern used to identify ranges or mixtures
#=================#
to_num <- function(dt, col_nm, na.values = c("Not available"),
                   match_pattern) {
  # Pull column values as vector
  cdat <- dt[[col_nm]]
  # Initialize output vector
  new <- vector(mode = "numeric", length = length(cdat))
  
  # Identify missing values
  na_match <- which(cdat %in% na.values)
  # Identify values to be split
  to_split <- grep(match_pattern, cdat)
  
  # Assign missing values as NA
  new[na_match] <- as.numeric(NA)
  # Split and return the minimum value
  new[to_split] <- sapply(strsplit(cdat[to_split], match_pattern), function(x) min(parse_number(x), na.rm = T))
  # Add in standard numeric values
  new[-c(na_match, to_split)] <- as.numeric(cdat[-c(na_match, to_split)])
  return(new)
}


# WEIGHT OF EVIDENCE SCORE -----
#=================#
# Derives WoE extrapolated clasifications and derives WoE score
# based on individual scoring schema.
#-----------------#
# `dt` = tibble
# `call_col` = Name of the column containing active/inactive calls
# `conc_col` = Name of the column containing numeric concentrations
# `dsa_col` = Name of the column containing numeric DSAs
# `dsa1_col` = Name of the column containing numeric DSA1+
# `group_col` = Name of the column with group identifiers (usually chemical identifier)
# `inactive_name` = The string or value in `call_col` corresponding to inactive calls
# `active_name` = The string or value in `call_col` corresponding to active calls
#=================#
hppt_woe_ec_indiv <- function(dt, call_col, conc_col, dsa_col, dsa1_col, group_col = NULL,
                              inactive_name = "Inactive", active_name = "Active") {
  classes_sorted <- c("NC", "NC/1B", "NC/1", "1B", "1B+", "POS", "1A-", "1A")
  woe_ec_scores <- c("NC" = 0, "NC/1B" = 0.5, "NC/1" = NA, "1B" = 1, "1B+" = 1.25, "POS" = 1.5, "1A-" = 1.75, "1A" = 2)
  dt <- dt %>%
    group_by({{ group_col }}) %>%
    mutate(
      ec = case_when(
        {{ call_col }} == inactive_name & is.na({{ conc_col }}) ~ "NC/1",
        {{ call_col }} == inactive_name & {{ conc_col }} < 25 & is.na({{ dsa_col }}) ~ "NC/1",
        {{ call_col }} == inactive_name & {{ conc_col }} < 25 & {{ dsa_col }} <= 375 ~ "NC/1",
        {{ call_col }} == inactive_name & {{ conc_col }} < 25 & {{ dsa_col }} > 375 ~ "NC/1B",
        {{ call_col }} == inactive_name & {{ conc_col }} >= 25 ~ "NC",
        {{ call_col }} == active_name & is.na(dsa1_new) ~ "POS",
        {{ call_col }} == active_name & {{ dsa1_col }} > 625 ~ "1B",
        {{ call_col }} == active_name & {{ dsa1_col }} > 500 & {{ dsa1_col }} <= 625 ~ "1B+",
        {{ call_col }} == active_name & {{ dsa1_col }} > 375 & {{ dsa1_col }} <= 500 ~ "1A-",
        {{ call_col }} == active_name & {{ dsa1_col }} <= 375 ~ "1A"
      ),
      ec = factor(ec, levels = classes_sorted),
      woe_score = woe_ec_scores[ec]
    ) %>%
    ungroup()
  return(dt)
}

#=================#
# Derives overall reference classification from overall WoE score
#-----------------#
# `dt` = tibble
# `group_col` = Name of the column with group identifiers (usually chemical identifier)
# `indiv_woe_score_col` = Column name containing the individual WoE scores.
#=================#
# Note: Here, we use true NA to indicate no data were available to derive
# WoE scores. As such, when the WoE score is NA, the extrapolated classification
# will also be NA because there is nothing to evaluate. "Not applicable" is used
# when the classification table specifies "na", indicating that there exist
# data to evaluate, but it is not informative.
#=================#
hppt_woe_class <- function(dt, group_col = NULL, indiv_woe_score_col = woe_score) {
  dt <- dt %>%
    group_by({{ group_col }}) %>%
    summarize(overall_woe_score = rnd(mean({{ indiv_woe_score_col }}, na.rm = T), digits = 2)) %>%
    ungroup() %>%
    mutate(
      overall_woe_score = ifelse(is.nan(overall_woe_score), as.character(NA), overall_woe_score),
      woe_ghs_bin = case_when(
        is.na(overall_woe_score) ~ as.character(NA),
        overall_woe_score <= 0.25 ~ "NC",
        overall_woe_score <= 0.75 ~ "Not applicable",
        overall_woe_score <= 2 ~ "1"
      ),
      woe_ghs_sub = case_when(
        is.na(overall_woe_score) ~ as.character(NA),
        overall_woe_score <= 0.25 ~ "NC",
        overall_woe_score <= 0.75 ~ "Not applicable",
        overall_woe_score <= 1.49 ~ "1B",
        overall_woe_score == 1.50 ~ "Not applicable",
        overall_woe_score <= 2 ~ "1A"
      ),
      woe_ghs_border = case_when(
        is.na(overall_woe_score) ~ as.character(NA),
        overall_woe_score <= 0.25 ~ "NC",
        overall_woe_score <= 0.75 ~ "NC/1B",
        overall_woe_score <= 1.25 ~ "1B",
        overall_woe_score <= 1.75 ~ "1",
        overall_woe_score <= 2 ~ "1A"
      )
    )
  return(dt)
}

# MEDIAN-LIKE LOCATION PARAMETER -----
#=================#
# Derives the MLLP value
#-----------------#
# middle_values : The extrapolated classification value(s) being evaluated
#-----------------#
# Returns a single MLLP value
#=================#
mllp_logic <- function(middle_values) {
  val_num <- na.omit(suppressWarnings(as.numeric(middle_values)))
  val_med <- median(val_num, na.rm = T)
  out <- case_when(
    !is.na(val_med) ~ as.character(val_med),
    any(middle_values == "POS") ~ "POS",
    any(middle_values == "NC/1B") ~ "NC/1B",
    any(middle_values == "NC") ~ "NC",
    all(middle_values == "NC/1") ~ "NC/1"
  )
  return(out)
}

#=================#
# Helper function to derive the GHS categorizations using the MLLP paradigm
#-----------------#
# mllp_bin : The MLLP value used for binary classification
# mllp_sub : The MLLP value used for sub and border classification
#-----------------#
# Returns the 3 GHS classifications
#=================#
mllp_cat <- function(mllp_bin, mllp_sub) {
  mllp_ghs_bin <- mllp_ghs_sub <- mllp_ghs_border <- NA
  mllp_bin_num <- suppressWarnings(as.numeric(mllp_bin))
  mllp_sub_num <- suppressWarnings(as.numeric(mllp_sub))
  
  mllp_ghs_bin <- case_when(
    is.na(mllp_bin) ~ as.character(NA),
    mllp_bin == "NC" ~ "NC",
    mllp_bin %in% c("NC/1B", "NC/1") ~ "Not applicable",
    mllp_bin == "POS" ~ "1",
    !is.na(mllp_bin_num) ~ "1"
  )
  
  mllp_ghs_sub <- case_when(
    is.na(mllp_sub) ~ as.character(NA),
    mllp_sub == "NC" ~ "NC",
    mllp_sub %in% c("NC/1B", "NC/1") ~ "Not applicable",
    !is.na(mllp_sub_num) & mllp_sub_num > 500 ~ "1B",
    !is.na(mllp_sub_num) & mllp_sub_num <= 500 ~ "1A"
  )
  
  mllp_ghs_border <- case_when(
    is.na(mllp_sub) ~ as.character(NA),
    mllp_sub == "NC" ~ "NC",
    mllp_sub == "NC/1" ~ "Not applicable",
    mllp_sub == "NC/1B" ~ "NC/1B",
    !is.na(mllp_sub_num) & mllp_sub_num > 625 ~ "1B",
    !is.na(mllp_sub_num) & mllp_sub_num > 375 ~ "1",
    !is.na(mllp_sub_num) & mllp_sub_num <= 375 ~ "1A"
  )
  
  out <- c(
    MLLP_GHS_bin = mllp_ghs_bin,
    MLLP_GHS_sub = mllp_ghs_sub,
    MLLP_GHS_border = mllp_ghs_border
  )
  return(out)
}

#=================#
# Derives MLLP values to be used for classification. This function takes in
# 3 vectors that should be equal length and sorted the same, so that
# values in the same position in each 3 vector correspond to the same record.
# The MLLP for binary classification uses "POS" for determining the median
# whereas sub and border classifications do not use "POS". Therefore,
# there will be two different MLLP values reported. One for binary (MLLP_Bin)
# and one for both sub and border (MLLP_sub).
#-----------------#
# dsa_vec : A vector of all DSA values for a given chemical
# dsa1_vec : A vector of all DSA1+ values for a given chemical
# ec_vec : A vector of all extrapolated classifications for a given chemical
#=================#
# Note, true NA is used for cases when a chemical only as ambiguous negative
# classifications, as these are not meant to be evaluated with MLLP.
#=================#
mllp_score <- function(dsa_vec, dsa1_vec, ec_vec) {
  # Sort the vectors by EC and DSA1+
  # ec_vec <- factor(ec_vec, levels = c("NC", "NC/1B", "NC/1", "1B", "1B+", "POS", "1A-", "1A"))
  ec_vec <- factor(ec_vec, levels = c("NC", "NC/1", "NC/1B", "1B", "1B+", "POS", "1A-", "1A"))
  new_ord <- order(ec_vec, dsa1_vec, decreasing = c(F, T))
  dsa_vec <- dsa_vec[new_ord]
  dsa1_vec <- dsa1_vec[new_ord]
  ec_vec <- ec_vec[new_ord]
  
  # Derive the median DSA1+
  pos_med <- median(dsa1_vec, na.rm = T)
  
  # Create a vector for evaluation. If a DSA1+ value exists, the value returned
  # is the DSA1+ value, otherwise it is the EC
  for_median <- ifelse(!is.na(dsa1_vec), as.character(dsa1_vec), as.character(ec_vec))
  
  # If the chemical only has ambiguous negative classifications, return NA
  if (all(ec_vec %in% c("NC/1", "NC/1B"))) {
    out <- c(MLLP_bin = as.character(NA), MLLP_sub = as.character(NA))
    # If the available individual test result outcomes are only NC or NC/1B,
    # the overall MLLP is NC. Added NC/1 to logic.
  } else if (all(ec_vec %in% c("NC", "NC/1B", "NC/1"))) {
    out <- c(MLLP_bin = "NC", MLLP_sub = "NC")
  } else {
    # Create logical vectors to determine whether values should be included
    # in the GHS classification scheme
    use_bin <- vector(mode = "logical", length = length(ec_vec))
    # For binary classification, use all positive results
    use_bin[ec_vec %in% c("1B", "1B+", "POS", "1A-", "1A")] <- T
    # For any negative results, compare to the median of the positive DSA1+ values
    use_bin[ec_vec %in% c("NC", "NC/1B", "NC/1") & (is.na(dsa_vec) | is.na(pos_med))] <- F
    use_bin[ec_vec %in% c("NC", "NC/1B", "NC/1") & dsa_vec < pos_med] <- F
    use_bin[ec_vec %in% c("NC", "NC/1B", "NC/1") & dsa_vec >= pos_med] <- T
    # For sub and border classifications, the ambiguous POS is not used.
    use_sub <- use_bin
    use_sub[ec_vec == "POS"] <- F
    
    # Subset the values for evaluation
    for_bin <- for_median[use_bin]
    n_bin <- length(for_bin)
    for_sub <- for_median[use_sub]
    n_sub <- length(for_sub)
    
    # If there are no values left to evaluate, return NA
    if (n_bin == 0) { # If n_bin is 0,
      out <- c(MLLP_bin = as.character(NA), MLLP_sub = as.character(NA))
      # out <- c(MLLP_bin = "Not applicable", MLLP_sub = "Not applicable")
      # If there is only one value to be evaluated, return that value as the MLLP
    } else if (n_bin == 1) {
      if (n_sub == 0) {
        # out <- c(MLLP_bin = for_bin, MLLP_sub = "Not applicable")
        out <- c(MLLP_bin = for_bin, MLLP_sub = as.character(NA))
      } else if (n_sub == 1) {
        out <- c(MLLP_bin = for_bin, MLLP_sub = for_sub)
      }
    } else {
      bin_middle <- get_median(for_bin)
      sub_middle <- get_median(for_sub)
      if (length(bin_middle) == 1) {
        out_bin <- bin_middle
      } else if (length(bin_middle) == 2) {
        out_bin <- mllp_logic(bin_middle)
      }
      if (length(sub_middle) == 1) {
        out_sub <- sub_middle
      } else if (length(sub_middle) == 2) {
        out_sub <- mllp_logic(sub_middle)
      }
      out <- c(MLLP_bin = out_bin, MLLP_sub = out_sub)
    }
  }
  cats <- mllp_cat(out["MLLP_bin"], out["MLLP_sub"])
  out <- c(out, cats)
  return(data.frame(t(out)))
}

#=================#
# Function to derive the MLLP scores and corresponding GHS classes
#-----------------#
# `dt` = tibble
# `dsa_col` = Name of the column containing numeric DSAs
# `dsa1_col` = Name of the column containing numeric DSA1+
# `ec_col` = Name of the column containing the individual extrapolated classes
# `group_col` = Name of the column with group identifiers (usually chemical identifier)
#=================#
mllp <- function(dt, dsa_col, dsa1_col, ec_col, group_col = NULL) {
  dt <- dt %>%
    group_by({{ group_col }}) %>%
    summarize(mllp_score(
      dsa_vec = {{ dsa_col }},
      dsa1_vec = {{ dsa1_col }},
      ec_vec = {{ ec_col }}
    )) %>%
    ungroup()
  return(dt)
}

# MEDIAN SENSITISATION POTENCY ESTIMATE -----
#=================#
# Derives the MSPE value
#-----------------#
# middle_values : The extrapolated classification value(s) being evaluated
#-----------------#
# Returns a single MSPE value
#=================#
mspe_logic <- function(middle_values) {
  val_num <- na.omit(suppressWarnings(as.numeric(middle_values)))
  val_med <- median(val_num, na.rm = T)
  out <- case_when(
    !is.na(val_med) ~ as.character(val_med),
    any(middle_values == "POS") ~ "POS",
    any(middle_values == "NC/1B") ~ "NC/1B",
    any(middle_values == "NC") ~ "NC"
  )
  return(out)
}

#=================#
# Helper function to derive the GHS categorizations using the MSPE paradigm
#-----------------#
# `mspe_val` : The MSPE value used for classification
#-----------------#
# Returns the 3 GHS classifications
#=================#
mspe_cat <- function(mspe_val) {
  mspe_ghs_bin <- mspe_ghs_sub <- mspe_ghs_border <- NA
  mspe_num <- suppressWarnings(as.numeric(mspe_val))
  
  mspe_ghs_bin <- case_when(
    is.na(mspe_val) ~ as.character(NA),
    mspe_val == "NC" ~ "NC",
    mspe_val == "NC/1B" ~ "Not applicable",
    !is.na(mspe_num) | mspe_val == "POS" ~ "1"
  )
  
  mspe_ghs_sub <- case_when(
    is.na(mspe_val) ~ as.character(NA),
    mspe_val == "NC" ~ "NC",
    mspe_val == "NC/1B" ~ "Not applicable",
    !is.na(mspe_num) & mspe_num > 500 ~ "1B",
    mspe_val == "POS" ~ "Not applicable",
    !is.na(mspe_num) & mspe_num <= 500 ~ "1A"
  )
  
  mspe_ghs_border <- case_when(
    is.na(mspe_val) ~ as.character(NA),
    mspe_val == "NC" ~ "NC",
    mspe_val == "NC/1B" ~ "NC/1B",
    !is.na(mspe_num) & mspe_num > 625 ~ "1B",
    !is.na(mspe_num) & mspe_num <= 375 ~ "1A",
    !is.na(mspe_num) & mspe_num <= 500 ~ "1",
    mspe_val == "POS" ~ "1"
  )
  
  out <- c(
    MSPE_GHS_bin = mspe_ghs_bin,
    MSPE_GHS_sub = mspe_ghs_sub,
    MSPE_GHS_border = mspe_ghs_border
  )
  return(out)
}

#=================#
# Derives MSPE values to be used for classification. This function takes in
# 3 vectors that should be equal length and sorted the same, so that
# values in the same position in each 3 vector correspond to the same record.
#-----------------#
# dsa_vec : A vector of all DSA values for a given chemical
# dsa1_vec : A vector of all DSA1+ values for a given chemical
# ec_vec : A vector of all extrapolated classifications for a given chemical
#=================#
mspe_score <- function(dsa_vec, dsa1_vec, ec_vec) {
  # Sort the vectors by EC and DSA1+
  ec_vec <- factor(ec_vec, levels = c("NC", "NC/1B", "NC/1", "1B", "1B+", "POS", "1A-", "1A"))
  new_ord <- order(ec_vec, dsa1_vec, decreasing = c(F, T))
  dsa_vec <- dsa_vec[new_ord]
  dsa1_vec <- dsa1_vec[new_ord]
  ec_vec <- ec_vec[new_ord]
  
  # Derive the median DSA1+
  pos_med <- median(dsa1_vec, na.rm = T)
  
  # Create a vector for evaluation. If a DSA1+ value exists, the value returned
  # is the DSA1+ value, otherwise it is the EC
  for_median <- ifelse(!is.na(dsa1_vec), as.character(dsa1_vec), as.character(ec_vec))
  
  # If the chemical only has ambiguous negative classifications, return NA
  if (all(ec_vec == "NC/1") | all(ec_vec == "NC/1B")) {
    out <- as.character(NA)
    # If there are one or more NC results and all other test outcomes are NC/1B, the MSPE is NC.
  } else if (all(ec_vec %in% c("NC", "NC/1B"))) {
    out <- "NC"
  } else {
    # Create logical vectors to determine whether values should be included
    # in the GHS classification scheme
    use_val <- vector(mode = "logical", length = length(ec_vec))
    # NC/1 test results were completely excluded from the assessment
    use_val[ec_vec == "NC/1"] <- F
    # Positive test results with a POS outcome (i.e. without an available DSA1+
    # value) are included when determining the position of the median
    use_val[ec_vec %in% c("1B", "1B+", "POS", "1A-", "1A")] <- T
    # Assuming the MSPE method follows MLLP method for filtering negative results
    use_val[ec_vec %in% c("NC", "NC/1B") & (is.na(dsa_vec) | is.na(pos_med))] <- F
    use_val[ec_vec %in% c("NC", "NC/1B") & dsa_vec < pos_med] <- F
    use_val[ec_vec %in% c("NC", "NC/1B") & dsa_vec >= pos_med] <- T
    
    # Subset the values for evaluation
    for_mspe <- for_median[use_val]
    n_mspe <- length(for_mspe)
    
    # Label whether the values need to be evaluated for equal 1A and 1B
    check_equal <- !is.na(pos_med) & any(ec_vec == "NC/1B") & all(ec_vec != "NC")
    
    # If there are no values left to evaluate, return NA
    if (n_mspe == 0) {
      out <- as.character(NA)
      # out <- "Not applicable"
      # If only one value, record the value
    } else if (n_mspe == 1) {
      out <- for_mspe
    } else if (check_equal) {
      # If there are one or more positive results in addition to one or more
      # NC/1B results, but there is no clear NC result, the median DSA1+ of the
      # positive results with numerical values is taken as the MSPE. However,
      # in all of these cases in which the number of 1A (incl. 1A-) study
      # results equals that of the 1B (incl. 1B+) results, the MSPE is POS.
      
      class_1a <- sum(ec_vec %in% c("1A-", "1A") & !is.na(dsa1_vec))
      class_1b <- sum(ec_vec %in% c("1B", "1B+") & !is.na(dsa1_vec))
      
      if (class_1a == class_1b) {
        out <- "POS"
      } else {
        for_mspe <- na.omit(dsa1_vec)
        val_middle <- get_median(for_mspe)
        if (length(val_middle) == 1) {
          out <- val_middle
        } else if (length(val_middle) == 2) {
          out <- mspe_logic(val_middle)
        }
      }
    } else {
      val_middle <- get_median(for_mspe)
      if (length(val_middle) == 1) {
        out <- val_middle
      } else if (length(val_middle) == 2) {
        out <- mspe_logic(val_middle)
      }
    }
  }
  cats <- mspe_cat(out)
  out <- c(MSPE = out, cats)
  return(data.frame(t(out)))
}

#=================#
# Function to derive the MSPE scores and corresponding GHS classes
#-----------------#
# `dt` = tibble
# `dsa_col` = Name of the column containing numeric DSAs
# `dsa1_col` = Name of the column containing numeric DSA1+
# `ec_col` = Name of the column containing the individual extrapolated classes
# `group_col` = Name of the column with group identifiers (usually chemical identifier)
#=================#
mspe <- function(dt, dsa_col, dsa1_col, ec_col, group_col = NULL) {
  dt %>%
    group_by({{ group_col }}) %>%
    summarize(mspe_score(
      dsa_vec = {{ dsa_col }},
      dsa1_vec = {{ dsa1_col }},
      ec_vec = {{ ec_col }}
    )) %>%
    ungroup()
}

# OVERALL WEIGHT OF EVIDENCE ----
#=================#
# Derives GHS classifications for all 3 methods
#-----------------#
# `dt` = tibble
# `call_col` = Name of the column containing active/inactive calls
# `conc_col` = Name of the column containing numeric concentrations
# `dsa_col` = Name of the column containing numeric DSAs
# `dsa1_col` = Name of the column containing numeric DSA1+
# `group_col` = Name of the column with group identifiers (usually chemical identifier)
# `inactive_name` = The string or value in `call_col` corresponding to inactive calls
# `active_name` = The string or value in `call_col` corresponding to active calls
#=================#
hppt_ghs_class <- function(dt, call_col, conc_col, dsa_col, dsa1_col,
                           group_col = NULL, inactive_name = "Inactive", active_name = "Active") {
  ec_indiv <- dt %>%
    group_by({{ group_col }}) %>%
    hppt_woe_ec_indiv(
      call_col = {{ call_col }},
      conc_col = {{ conc_col }},
      dsa_col = {{ dsa_col }},
      dsa1_col = {{ dsa1_col }},
      group_col = {{ group_col }},
      inactive_name = inactive_name,
      active_name = active_name
    )
  
  woe_class <- hppt_woe_class(
    dt = ec_indiv,
    group_col = {{ group_col }},
    indiv_woe_score_col = woe_score
  )
  
  m_class <- ec_indiv %>%
    group_by({{ group_col }}) %>%
    summarize(
      mllp_score(
        dsa_vec = {{ dsa_col }},
        dsa1_vec = {{ dsa1_col }},
        ec_vec = ec
      ),
      mspe_score(
        dsa_vec = {{ dsa_col }},
        dsa1_vec = {{ dsa1_col }},
        ec_vec = ec
      )
    )
  
  by_col <- as_label(enquo(group_col))
  out <- full_join(woe_class, m_class, by = by_col)
  out <- list(
    ec_indiv = ec_indiv,
    overall_classes = out
  )
  return(out)
}

#=================#
# Derives overall ghs classifications
#-----------------#
# `hppt_ghs_class_list` = list of length 2, returned from `hppt_ghs_class`
# `group_col` = Name of the column with group identifiers (usually chemical identifier)
#=================#
hppt_overall_classification <- function(hppt_ghs_class_list, group_col) {
  # Convert column names to lowercase
  names(hppt_ghs_class_list$overall_classes) <- tolower(names(hppt_ghs_class_list$overall_classes))
  group_col <- as_label(enquo(group_col))
  # For each substance, count the number of tests and tally the 
  # extrapolated classifications
  overall_classes <- hppt_ghs_class_list$ec_indiv %>%
    group_by(across(all_of(group_col))) %>%
    mutate(N = n()) %>%
    group_by(across(all_of(group_col)), N, ec) %>%
    summarize(n_class = n(), .groups = "keep") %>%
    ungroup() %>%
    pivot_wider(names_from = ec, values_from = n_class, values_fill = 0) %>%
    full_join(hppt_ghs_class_list$overall_classes, by = group_col)
  
  # If a substance has the same classification for the three methods, that
  # classification is set as the overall classification. Otherwise, no
  # overall classification is defined for the substance.
  ctype <- paste("ghs", c("bin", "sub", "border"), sep = "_")
  
  for (i in ctype) {
    coltmp <- grep(i, names(overall_classes), value = T)
    overall_classes <- overall_classes %>%
      rowwise() %>%
      mutate_at(coltmp, list(~ifelse(. == "Not applicable", NA, .))) %>%
      mutate(
        tmp_list = list(c(!!!syms(coltmp))),
        tmp_cname = case_when(
          all(is.na(tmp_list)) ~ as.character(NA),
          length(unique(na.omit(tmp_list))) == 1 ~ na.omit(tmp_list)[1],
          T ~ as.character(NA)
        )
      ) %>%
      select(-tmp_list) %>%
      rename_with(~ paste0("overall_", i), tmp_cname) %>%
      ungroup()
  }
  
  out <- overall_classes %>%
    select(all_of(group_col),
           starts_with("overall_ghs"),
           mllp_value_bin = mllp_bin,
           mllp_value_sub = mllp_sub,
           mllp_ghs_bin, mllp_ghs_sub, mllp_ghs_border,
           mspe_value = mspe,
           mspe_ghs_bin, mspe_ghs_sub, mspe_ghs_border,
           woe_value = overall_woe_score,
           woe_ghs_bin, woe_ghs_sub, woe_ghs_border,
           total_tests = N,
           levels(hppt_ghs_class_list$ec_indiv$ec)
    )
  
  return(out)
}

#=================#
# Resolves discordant GHS sub and GHS border outcomes
#-----------------#
# `ec_indiv` = tibble output from `hppt_ghs_class()` containing individual 
#    extrapolated classifications
# `repro_dsa1` = reproducibility tibble output from `hppt_repro()` for DSA1+
# `repro_dsa05` = reproducibility tibble output from `hppt_repro()` for DSA5%
# `ghs_sub` = logical, whether to resolve GHS sub outcomes
# `ghs_border` = logical, whether to resolve GHS border outcomes
#=================#
hppt_resolve_overall <- function(dsa1_overall = NULL, dsa05_overall = NULL, ghs_sub = T, ghs_border = T, group_col) {
  doDSA1 <- !is.null(dsa1_overall)
  doDSA05 <- !is.null(dsa05_overall)
  
  if (ghs_sub) {
    sub_cols <- c("woe_ghs_sub", "mllp_ghs_sub", "mspe_ghs_sub")
    if (doDSA05) {
      dsa05_discord <- apply(dsa05_overall[sub_cols], 1, function(x) length(unique(na.omit(x))) > 1)
      dsa05_1a <- apply(dsa05_overall[c("1A", "1A-")], 1, function(x) any(x > 0))
      
      dsa05_overall[dsa05_discord & dsa05_1a,"overall_ghs_sub"] <- "1A"
    }
    
    if (doDSA1) {
      dsa1_discord <- apply(dsa1_overall[sub_cols], 1, function(x) length(unique(na.omit(x))) > 1)
      dsa1_1a <- apply(dsa1_overall[c("1A", "1A-")], 1, function(x) any(x > 0))
      
      dsa1_overall[dsa1_discord & dsa1_1a,"overall_ghs_sub"] <- "1A"
      
      if (doDSA05) {
        dsa1_discord_05 <- dsa1_discord & !dsa1_1a
        if (any(dsa1_discord_05)) {
          idx <- which(dsa1_discord_05)
          for (i in idx) {
            newOA <- dsa05_overall %>% filter(if_all(group_col, ~ . == i)) %>% pull(overall_ghs_sub)
            print(newOA)
            if (!is.na(newOA)) {
              dsa1_overall[i,"overall_ghs_sub"] <- newOA
            }
          }
        }
      }
      
    }
  }
  
  if (ghs_border) {
    border_cols <- c("woe_ghs_border", "mllp_ghs_border", "mspe_ghs_border")
    if (doDSA05) {
      dsa05_overall <- dsa05_overall %>%
        rowwise() %>%
        mutate(
          tmp_list = list(c(!!!syms(border_cols))),
          tmp_border = length(unique(na.omit(tmp_list))),
          overall_ghs_border = case_when(
            tmp_border <= 1 ~ overall_ghs_border,
            tmp_border == 3 ~ case_when(
              all(c("1A", "1", "1B") %in% tmp_list) ~ "1",
              all(c("1", "1B", "NC/1B") %in% tmp_list) ~ "1B",
              all(c("1B", "NC/1B", "NC") %in% tmp_list) ~ "NC/1B"
            ),
            tmp_border == 2 ~ case_when(
              all(c("1A", "1") %in% tmp_list) ~ "1A",
              all(c("1A", "1B") %in% tmp_list) ~ "1",
              all(c("1", "1B") %in% tmp_list) ~ "1B",
              all(c("1", "NC/1B") %in% tmp_list) ~ "1B",
              all(c("1B", "NC/1B") %in% tmp_list) ~ "1B",
              all(c("1B", "NC") %in% tmp_list) ~ "NC/1B",
              all(c("NC/1B", "NC") %in% tmp_list) ~ "NC"
            )
          )
        ) %>%
        ungroup() %>%
        select(-tmp_list, -tmp_border)
    }
    
    if (doDSA1) {
      dsa1_overall <- dsa1_overall %>%
        rowwise() %>%
        mutate(
          tmp_list = list(c(!!!syms(border_cols))),
          tmp_border = length(unique(na.omit(tmp_list))),
          overall_ghs_border = case_when(
            tmp_border <= 1 ~ overall_ghs_border,
            tmp_border == 3 ~ case_when(
              all(c("1A", "1", "1B") %in% tmp_list) ~ "1",
              all(c("1", "1B", "NC/1B") %in% tmp_list) ~ "1B",
              all(c("1B", "NC/1B", "NC") %in% tmp_list) ~ "NC/1B"
            ),
            tmp_border == 2 ~ case_when(
              all(c("1A", "1") %in% tmp_list) ~ "1A",
              all(c("1A", "1B") %in% tmp_list) ~ "1",
              all(c("1", "1B") %in% tmp_list) ~ "1B",
              all(c("1", "NC/1B") %in% tmp_list) ~ "1B",
              all(c("1B", "NC/1B") %in% tmp_list) ~ "1B",
              all(c("1B", "NC") %in% tmp_list) ~ "NC/1B",
              all(c("NC/1B", "NC") %in% tmp_list) ~ "NC"
            )
          )
        ) %>%
        ungroup() %>%
        select(-tmp_list, -tmp_border)
    }
  }
  
  return(list(dsa1_overall = dsa1_overall, dsa05_overall = dsa05_overall))
}

#=================#
# Derives reproducibility for GHS_BIN and GHS_SUB. 
#-----------------#
# `hppt_ghs_table` = table with overall GHS classifications, returned from `hppt_overall_classification`
#=================#
hppt_repro <- function(hppt_ghs_table, group_col) {
  # Reproducibility is calculated for ghs_bin and ghs_sub. Reproducibility of 
  # GHS_BIN is the fraction of all HPPT results for a substance with an
  # unambiguous classification of 1 or NC that is equal to the overall 
  # binary classification. Reproducibility of GHS_SUB is the fraction of 
  # all HPPT results for a substance with an unambiguous classification (1A, 1B, NC)
  # that is equal to the overall sub classification.
  
  # GHS BIN
  hppt_ghs_table$n_bin <- rowSums(hppt_ghs_table[,c("NC", "1B", "1B+", "POS","1A-", "1A")], na.rm = T)
  bin1 <- which(hppt_ghs_table$overall_ghs_bin == "1")
  bin0 <- which(hppt_ghs_table$overall_ghs_bin == "NC")
  
  hppt_ghs_table$repro_ghs_bin <- NA
  hppt_ghs_table$repro_ghs_bin[bin1] <- 100 * rowSums(hppt_ghs_table[bin1, c("1B", "1B+", "POS","1A-", "1A")])/hppt_ghs_table[bin1,"n_bin", drop = T]
  hppt_ghs_table$repro_ghs_bin[bin0] <- 100 * (hppt_ghs_table[bin0, "NC", drop = T]/hppt_ghs_table[bin0, "n_bin", drop = T])
  
  # GHS SUB
  sub1a <- which(hppt_ghs_table$overall_ghs_sub == "1A")
  sub1b <- which(hppt_ghs_table$overall_ghs_sub == "1B")
  subnc <- which(hppt_ghs_table$overall_ghs_sub == "NC")
  
  hppt_ghs_table$n_sub <- rowSums(hppt_ghs_table[,c("NC", "1B", "1B+","1A-", "1A")], na.rm = T)
  hppt_ghs_table$n_sub[sub1a] <- hppt_ghs_table$n_sub[sub1a] + hppt_ghs_table[["NC/1B"]][sub1a]
  
  hppt_ghs_table$repro_ghs_sub <- NA
  hppt_ghs_table$repro_ghs_sub[sub1a] <- 100 * rowSums(hppt_ghs_table[sub1a, c("1A-", "1A")])/hppt_ghs_table[sub1a,"n_sub", drop = T]
  hppt_ghs_table$repro_ghs_sub[sub1b] <- 100 * rowSums(hppt_ghs_table[sub1b, c("1B+", "1B")])/hppt_ghs_table[sub1b,"n_sub", drop = T]
  hppt_ghs_table$repro_ghs_sub[subnc] <- 100 * (hppt_ghs_table[subnc, "NC", drop = T]/hppt_ghs_table[subnc,"n_sub", drop = T])
  
  hppt_ghs_table %>%
    select(
      {{ group_col }},
      starts_with("overall_ghs"),
      starts_with("repro_"),
      starts_with("mllp_"),
      starts_with("mspe_"),
      starts_with("woe_"),
      total_tests,
      total_tests_repro_bin = n_bin,
      total_tests_repro_sub = n_sub,
      all_of(c("NC", "NC/1B", "NC/1", "1B", "1B+", "POS","1A-", "1A"))
    )
}
```

# 2 Data Clean

First, the data are read in as a tibble with 2,277 rows and 31
columns.


```
fname <- "2022.08.10_HPPT_databasev2.xlsx"
dat <- readxl::read_excel(fname, sheet = 1, col_types = "text", na = c("", "na"))
dim(dat)
```


```
[1] 2277   31
```


For data cleaning, we use the following procedure:

1. Remove test results with **RRS = 5**
2. Assign unique chemical IDs based on CASRN when available, and
   ‘Synonyms’ column otherwise.
3. For coding purposes, columns ‘Call’, ‘Conc.(%)’, ‘DSA.(μg/cm2)’,
   ‘DSA1+.(μg/cm2)’, and ‘DSA05.(μg/cm2)’ are renamed to ‘pn’, ‘conc’,
   ‘dsa’, ‘dsa1’, and ‘dsa05’, respectively.
4. The columns conc, dsa, dsa1, and dsa05 are converted to numeric. If
   multiple values are given (e.g., range, mixture), the minimum numeric
   value is recorded and used for classification.

The data are filtered to keep records where the Relative Reliability
Score (RRS) < 5.


```
dat2 <- dat %>% filter(RRS != "5")
dim(dat2)
```


```
[1] 2255   31
```


How many unique, non-missing CASRN are in the database?


```
dat2 %>%
  filter(CASRN != "Not available") %>%
  pull(CASRN) %>%
  unique() %>%
  length()
```


```
[1] 1148
```


Among the substances without a CASRN, how many unique names are
there?


```
dat2 %>%
  filter(CASRN == "Not available") %>%
  pull(Synonyms) %>%
  unique() %>%
  length()
```


```
[1] 218
```


There are 1148 unique CASRNs in the data. Among records with no
CASRN, there are 218 unique names. The 2255 test results with RRS < 5
relate to a total of 1366 substances with a unique ID.

Unique substance IDs are created for each substance using the CASRN
and Synonyms.


```
dat2 <- dat2 %>%
  mutate(cid = ifelse(CASRN == "Not available", Synonyms, CASRN))
(n_cid <- length(unique(dat2$cid)))
```


```
[1] 1366
```


The data are trimmed to keep only columns that are needed. Columns
are renamed.


```
dat2 <- dat2 %>%
  select(
    all_of(
      c("Record.No",
        "cid",
        dsa = "DSA.(μg/cm2)",
        pn = "Call",
        conc = "Conc.(%)",
        dsa1 = "DSA1+.(μg/cm2)",
        dsa05 = "DSA05.(μg/cm2)"))
  )
```


Set up example row index


```
sampleCids <- c("1117-55-1", "1786-08-9", "85-91-6", "6969-49-9", "106-24-1", "106-26-3", "64-17-5", "150-86-7", "16409-43-1", "150-13-0", "56-75-7", "50-53-3", "2442-10-6", "763-35-9", "3374-22-9", "57378-68-4", "8003-05-2", "823-22-3", "116-26-7", "302-01-2", "617-54-9", "40607-48-5", "112-72-1", "54464-57-2", "28219-61-6")
example_rows_indiv <- sort(which(dat2$cid %in% sampleCids))
example_rows_group <- sort(which(unique(dat2$cid) %in% sampleCids))

indiv_cols <- c("cid", "dsa_new", "conc_new", "dsa1_new", "dsa05_new", "pn", "woe_score", "ec")
```


The function requires that the following columns be numeric.

- Concentration (%) - `conc`
- Dose per skin area (μg/cm2) - `dsa`
- Hypothetical dose per skin area resulting in one test subject with a
  positive test result under the conditions of the test
  (μg/cm2) - `dsa1+`

The classifications will also be derived using the hypothetical dose
per skin area resulting in 5% incidence under the conditions of the test
(μg/cm2) - `dsa05`- in place of
`dsa1+`.

Numeric conversion is performed using the sourced function
`to_num`.


```
dat2$conc_new <- to_num(dat2, "conc",
                        na.values = c("Not available"),
                        match_pattern = "to|and"
)
dat2$dsa_new <- to_num(dat2, "dsa",
                       na.values = "Not available",
                       match_pattern = "-"
)
dat2$dsa1_new <- to_num(dat2, "dsa1",
                        na.values = c("Not applicable", "Not available"),
                        match_pattern = "to"
)
dat2$dsa05_new <- to_num(dat2, "dsa05",
                         na.values = c("Not applicable", "Not available"),
                         match_pattern = "to"
)
```

# 3 Classification

## 3.1 Individual Methods

We use the function sourced `hppt_ghs_class` to derive GHS
classifications using 3 methods:

1. Weight-of-Evidence Score (WoE)
2. Median-Like Location Parameter (MLLP)
3. Median Sensitization Potency Estimate (MSPE)

The function also returns the individual extrapolated classifications
used to derive the GHS classifications.

### 3.1.1 DSA1+


```
dsa1_class <- hppt_ghs_class(
  dt = dat2,
  call_col = pn, # column containing active/inactive calls
  conc_col = conc_new,
  dsa_col = dsa_new,
  dsa1_col = dsa1_new,
  group_col = cid,
  inactive_name = "Inactive",
  active_name = "Active"
)
```


The first list item of the output contains the results for each
individual method for each of the chemicals. The Weight of Evidence
Score, MLLP, and MSPE use the individual ECs to classify GHS
categories.


```
dsa1_class$overall_classes[example_rows_group,]
```


The second list item of the output contains the individual record WoE
scores and the ECs for each record.


```
dsa1_class$ec_indiv[example_rows_indiv,indiv_cols]
```

### 3.1.2 DSA5%

Classifications are derived using the DSA05 value in place of
DSA1+.


```
dsa05_class <- hppt_ghs_class(
  dt = dat2,
  call_col = pn, # column containing active/inactive calls
  conc_col = conc_new,
  dsa_col = dsa_new,
  dsa1_col = dsa05_new,
  group_col = cid,
  inactive_name = "Inactive",
  active_name = "Active"
)
```


```
dsa05_class$overall_classes[example_rows_group,]
```


```
dsa05_class$ec_indiv[example_rows_indiv,indiv_cols]
```

## 3.2 Overall Weight of Evidence

We use the function `hppt_overall_classification` to
assign overall weight of evidence GHS clasifications. If the MLLP, MSPE,
and WoE Score outcomes are concordant, then that is the overall GHS
classification.

### 3.2.1 DSA1+

For each chemical, the output shows the individual method outcomes, a
summary of the individual ECs, and the overall GHS classifications.


```
dsa1_overall <- hppt_overall_classification(hppt_ghs_class_list = dsa1_class, group_col = cid)
dsa1_overall[example_rows_group,]
```

### 3.2.2 DSA5%


```
dsa05_overall <- hppt_overall_classification(dsa05_class, cid)
dsa05_overall[example_rows_group,]
```

### 3.2.3 Resolving Discordance

When the three approaches disagree for the GHS outcomes,
`hppt_overall_classification` does not assign a value. For
these chemicals, the OECD EG DASS evaluated the test results and applied
rule-guided expert judgement to define a consensus for GHSSUB
and GHSBORDER. The function `hppt_resolve_overall`
applies these rules where applicable.

Save the values to compare before and after:


```
sub_cols <- c("woe_ghs_sub", "mllp_ghs_sub", "mspe_ghs_sub")

dsa1_sub_discord <- apply(dsa1_overall[sub_cols], 1, function(x) length(unique(na.omit(x))) > 1)
dsa1_sub_discord_data <- dsa1_overall[dsa1_sub_discord,]

dsa05_sub_discord <- apply(dsa05_overall[sub_cols], 1, function(x) length(unique(na.omit(x))) > 1)
dsa05_sub_discord_data <- dsa05_overall[dsa05_sub_discord,]

border_cols <- c("woe_ghs_border", "mllp_ghs_border", "mspe_ghs_border")
dsa1_border_discord <- apply(dsa1_overall[border_cols], 1, function(x) length(unique(na.omit(x))) > 1)
dsa1_border_discord_data <- dsa1_overall[dsa1_border_discord,]

dsa05_border_discord <- apply(dsa05_overall[border_cols], 1, function(x) length(unique(na.omit(x))) > 1)
dsa05_border_discord_data <- dsa05_overall[dsa05_border_discord,]
```


```
resolve_out <- hppt_resolve_overall(
  dsa1_overall = dsa1_overall,
  dsa05_overall = dsa05_overall,
  group_col = "cid"
)
dsa1_overall <- resolve_out$dsa1_overall
dsa05_overall <- resolve_out$dsa05_overall
```


#### 3.2.3.1 GHS SUB

##### 3.2.3.1.1 DSA1+

Original:


```
sub_cols <- c("cid", "overall_ghs_sub", "mllp_ghs_sub", "mspe_ghs_sub", "woe_ghs_sub", "1A-", "1A")
dsa1_sub_discord_data[,sub_cols]
```


Rule Based:


```
dsa1_overall[dsa1_sub_discord,sub_cols]
```

##### 3.2.3.1.2 DSA5%

Original:


```
dsa05_sub_discord_data[,sub_cols]
```


Rule Based:


```
dsa05_overall[dsa05_sub_discord,sub_cols]
```

#### 3.2.3.2 GHS BORDER

##### 3.2.3.2.1 DSA1+

Original:


```
border_cols <- c("cid", "overall_ghs_border", "mllp_ghs_border", "mspe_ghs_border", "woe_ghs_border", "1A-", "1A")
dsa1_border_discord_data[,border_cols]
```


Rule Based:


```
dsa1_overall[dsa1_border_discord,border_cols]
```

##### 3.2.3.2.2 DSA5%

Original:


```
dsa05_border_discord_data[,border_cols]
```


Rule Based:


```
dsa05_overall[dsa05_border_discord,border_cols]
```

# 4 Reproducibility

## 4.1 GHS BIN

### 4.1.1 DSA1+


```
dsa1_repro <- hppt_repro(dsa1_overall, cid)
dsa1_repro[example_rows_group,c("cid", "overall_ghs_bin", "repro_ghs_bin", "total_tests_repro_bin", "NC", "1B", "1B+", "POS", "1A-", "1A")] %>%
  filter(!is.na(repro_ghs_bin))
```

### 4.1.2 DSA5%


```
dsa05_repro <- hppt_repro(dsa05_overall, cid)
dsa05_repro[example_rows_group,c("cid", "overall_ghs_bin", "repro_ghs_bin", "total_tests_repro_bin", "NC", "1B", "1B+", "POS", "1A-", "1A")] %>%
  filter(!is.na(repro_ghs_bin))
```

## 4.2 GHS SUB

### 4.2.1 DSA1+


```
dsa1_repro[example_rows_group,c("cid", "overall_ghs_sub", "repro_ghs_sub", "total_tests_repro_sub", "NC", "1B", "1B+", "1A-", "1A")] %>%
  filter(!is.na(repro_ghs_sub))
```

### 4.2.2 DSA5%


```
dsa05_repro[example_rows_group,c("cid", "overall_ghs_sub", "repro_ghs_sub", "total_tests_repro_sub", "NC", "1B", "1B+", "1A-", "1A")] %>%
  filter(!is.na(repro_ghs_sub))
```

# 5 Example: Penicillin G (CASRN 61-33-6/EC 200-506-3)

## 5.1 DSA1+

Distribution of individual extrapolated classifications based on
DSA1+:


```
dsa1_class$ec_indiv %>% 
  filter(cid == "61-33-6") %>%
  group_by(ec) %>%
  tally()
```


Results from individual methods:


```
dsa1_class$overall_classes %>%
  filter(cid == "61-33-6") %>%
  pivot_longer(cols = -cid)
```


Overall weight of evidence classification and reproducibility:


```
dsa1_repro %>%
  filter(cid == "61-33-6") %>%
  select(cid, starts_with("overall"), starts_with("repro")) %>%
  pivot_longer(cols = -cid, values_transform = as.character)
```

## 5.2 DSA5%

Distribution of individual extrapolated classifications based on
DSA5%:


```
dsa05_class$ec_indiv %>% 
  filter(cid == "61-33-6") %>%
  group_by(ec) %>%
  tally()
```


Results from individual methods:


```
dsa05_class$overall_classes %>%
  filter(cid == "61-33-6") %>%
  pivot_longer(cols = -cid)
```


Overall weight of evidence classification and reproducibility:


```
dsa05_repro %>%
  filter(cid == "61-33-6") %>%
  select(cid, starts_with("overall"), starts_with("repro")) %>%
  pivot_longer(cols = -cid, values_transform = as.character)
```

# 6 Save Output

Add identifiers back.


```
dict <- full_join(
  select(dat, Record.No, Synonyms, CASRN, EC.No),
  select(dat2, Record.No, cid),
  by = "Record.No"
)

dsa1_class$ec_indiv <- right_join(
  dict, dsa1_class$ec_indiv, by = c("cid", "Record.No")
) %>%
  select(-cid)

dsa05_class$ec_indiv <- right_join(
  dict, dsa05_class$ec_indiv, by = c("cid", "Record.No")) %>%
  select(-cid)

dict <- dict %>%
  select(-Record.No) %>%
  distinct() %>%
  group_by(CASRN, EC.No, cid) %>%
  mutate(Synonyms = gsub("[[:cntrl:]]+", " ", Synonyms)) %>%
  summarize(Synonyms = paste(unique(unlist(strsplit(Synonyms, "; "))), collapse = "; "),
            .groups = "keep") %>%
  ungroup() %>%
  relocate(Synonyms)

dsa1_class$overall_classes <- right_join(dict, dsa1_class$overall_classes, by = "cid") %>%
  select(-cid)
dsa1_repro <- right_join(dict, dsa1_repro, by = "cid") %>% select(-cid)
dsa05_class$overall_classes <- right_join(dict, dsa05_class$overall_classes, by = "cid") %>%
  select(-cid)
dsa05_repro <- right_join(dict, dsa05_repro, by = "cid") %>% select(-cid)
```


```
library(openxlsx)
fname1 <- paste0(format(Sys.time(), "%Y%m%d-%H%M-"), "-hpptClass-DSA1plus.xlsx")
wb <- createWorkbook()
addWorksheet(wb, sheetName = "IndividualECs")
writeData(wb, "IndividualECs", dsa1_class$ec_indiv, keepNA = T, na.string = "na")
addWorksheet(wb, sheetName = "GHSClassPred")
writeData(wb, "GHSClassPred", dsa1_class$overall_classes, keepNA = T, na.string = "na")
addWorksheet(wb, sheetName = "Reproducibility")
writeData(wb, "Reproducibility", dsa1_repro, keepNA = T, na.string = "na")
saveWorkbook(wb, fname1)

fname2 <- paste0(format(Sys.time(), "%Y%m%d-%H%M-"), "-hpptClass-DSA5perc.xlsx")
wb <- createWorkbook()
addWorksheet(wb, sheetName = "IndividualECs")
writeData(wb, "IndividualECs", dsa05_class$ec_indiv, keepNA = T, na.string = "na")
addWorksheet(wb, sheetName = "GHSClassPred")
writeData(wb, "GHSClassPred", dsa05_class$overall_classes, keepNA = T, na.string = "na")
addWorksheet(wb, sheetName = "Reproducibility")
writeData(wb, "Reproducibility", dsa05_repro, keepNA = T, na.string = "na")
saveWorkbook(wb, fname2)
```

# 7 Session Info


```
sessionInfo()
```


```
R version 4.2.0 (2022-04-22 ucrt)
Platform: x86_64-w64-mingw32/x64 (64-bit)
Running under: Windows 10 x64 (build 19044)

Matrix products: default

locale:
[1] LC_COLLATE=English_United States.utf8  LC_CTYPE=English_United States.utf8   
[3] LC_MONETARY=English_United States.utf8 LC_NUMERIC=C                          
[5] LC_TIME=English_United States.utf8    

attached base packages:
[1] stats     graphics  grDevices utils     datasets  methods   base     

other attached packages:
[1] tidyr_1.3.0      readr_2.1.4      dplyr_1.1.3      openxlsx_4.2.5.1 knitr_1.40      

loaded via a namespace (and not attached):
 [1] zip_2.3.0         Rcpp_1.0.11       cellranger_1.1.0  compiler_4.2.0   
 [5] pillar_1.9.0      later_1.3.1       tools_4.2.0       digest_0.6.31    
 [9] evaluate_0.22     lifecycle_1.0.3   tibble_3.2.1      pkgconfig_2.0.3  
[13] rlang_1.1.1       shiny_1.7.5       cli_3.6.1         rstudioapi_0.15.0
[17] writexl_1.4.2     yaml_2.3.7        xfun_0.40         fastmap_1.1.1    
[21] withr_2.5.1       hms_1.1.3         generics_0.1.3    vctrs_0.6.3      
[25] tidyselect_1.2.0  rprojroot_2.0.3   glue_1.6.2        R6_2.5.1         
[29] fansi_1.0.4       readxl_1.4.3      rmarkdown_2.17    purrr_1.0.2      
[33] tzdb_0.4.0        magrittr_2.0.3    promises_1.2.1    ellipsis_0.3.2   
[37] htmltools_0.5.6   mime_0.12         xtable_1.8-4      httpuv_1.6.11    
[41] utf8_1.2.3        stringi_1.7.12    miniUI_0.1.1.1    viewxl_0.1.4
```

LS0tDQp0aXRsZTogIlVzaW5nIEhQUFQgRGF0YSB0byBDbGFzc2lmeSBDaGVtaWNhbHMgd2l0aCBSZXNwZWN0IHRvIFRoZWlyIFNraW4gU2Vuc2l0aXNhdGlvbiBQb3RlbnRpYWwiDQphdXRob3I6ICJLaW0gVG8iDQpkYXRlOiAiYHIgZm9ybWF0KFN5cy50aW1lKCksICclZCAlQiwgJVknKWAiDQpvdXRwdXQ6IA0KICBodG1sX25vdGVib29rOg0KICAgIGRmX3ByaW50OiBwYWdlZA0KICAgIG51bWJlcl9zZWN0aW9uczogdHJ1ZQ0KICAgIHRvYzogdHJ1ZQ0KICAgIHRvY19mbG9hdDoNCiAgICAgIGNvbGxhcHNlZDogZmFsc2UNCi0tLQ0KDQpgYGB7Y3NzLCBlY2hvPUZBTFNFfQ0KaDEgew0KICBmb250LXNpemU6IDJlbTsNCn0NCg0KaDIgew0KICBmb250LXNpemU6IDEuNzVlbTsNCn0NCg0KaHIgew0KICB3aWR0aDogNTAlOw0KfQ0KDQoucmVmU2V0IHsNCiAgZm9udC1zaXplOiAwLjhlbTsNCiAgbGlzdC1zdHlsZS10eXBlOiBub25lOw0KICBwYWRkaW5nLWlubGluZS1zdGFydDogdW5zZXQ7DQp9DQoNCi5zY3JvbGwtbG9uZyB7DQogIG1heC1oZWlnaHQ6IDc1ZW07DQogIG92ZXJmbG93LXk6IGF1dG87DQogIGJhY2tncm91bmQtY29sb3I6IGluaGVyaXQ7DQp9DQpgYGANCg0KYGBge3Igc2V0dXAsIGVjaG8gPSBGfQ0KbGlicmFyeShrbml0cikNCmxpYnJhcnkob3Blbnhsc3gpDQpgYGANCg0KVGhlIE9yZ2FuaXphdGlvbiBmb3IgRWNvbm9taWMgQ28tb3BlcmF0aW9uIGFuZCBEZXZlbG9wbWVudCAoT0VDRCkgRXhwZXJ0IEdyb3VwIG9uIERlZmluZWQgQXBwcm9hY2hlcyBmb3IgU2tpbiBTZW5zaXRpemF0aW9uIChFRyBEQVNTKSBjdXJhdGVkIGEgSHVtYW4gUHJlZGljdGl2ZSBQYXRjaCBUZXN0IChIUFBUKSBkYXRhYmFzZSBhcyBhIHJlZmVyZW5jZSBzZXQgZm9yIGV2YWx1YXRpbmcgREFTUyBbMV0uIFRoZSBodW1hbiBkYXRhIHN1Yi1ncm91cCAoSERTRykgb2YgdGhlIE9FQ0QgRUcgREFTUyBldmFsdWF0ZWQgdGhlIEhQUFQgZGF0YSBmb3IgY2xhc3NpZmljYXRpb24gb2YgY2hlbWljYWxzIGFzIHNraW4gc2Vuc2l0aXplcnMgdW5kZXIgdGhlIFVuaXRlZCBOYXRpb25zJyBHbG9iYWxseSBIYXJtb25pemVkIFN5c3RlbSBvZiBDbGFzc2lmaWNhdGlvbiBhbmQgTGFiZWxsaW5nIG9mIENoZW1pY2FscyAoR0hTKSBbMl0uIFRoZSBIRFNHIGRldmVsb3BlZCBhIG1vZGlmaWVkIGNsYXNzaWZpY2F0aW9uIGFwcHJvYWNoIGJhc2VkIG9uIHR3byBleHRyYXBvbGF0ZWQgcmVzcG9uc2UgdmFsdWVzOg0KDQotICAgRFNBMSsgLSB0aGUgaHlwb3RoZXRpY2FsIGRvc2UtcGVyLXNraW4tYXJlYSB0aGF0IHNlbnNpdGl6ZXMgZXhhY3RseSBvbmUgdGVzdCBzdWJqZWN0DQotICAgRFNBNSUgLSB0aGUgaHlwb3RoZXRpY2FsIGRvc2UtcGVyLXNraW4tYXJlYSB0aGF0IHNlbnNpdGl6ZXMgNSUgb2YgdGVzdCBzdWJqZWN0cw0KDQpBIHdlaWdodC1vZi1ldmlkZW5jZSBhcHByb2FjaCB3YXMgdGhlbiBkZXZlbG9wZWQgdG8gY29tYmluZSBtdWx0aXBsZSBIUFBUIHJlc3VsdHMgZm9yIGEgY2hlbWljYWwgaW50byBhbiBvdmVyYWxsIEdIUyBjbGFzc2lmaWNhdGlvbi4NCg0KVGhpcyBSIG5vdGVib29rIGRlbW9uc3RyYXRlcyB0aGUgYXBwbGljYXRpb24gb2YgdGhlIG1vZGlmaWVkIGNsYXNzaWZpY2F0aW9uIGFuZCB3ZWlnaHQtb2YtZXZpZGVuY2UgYXBwcm9hY2hlcyB0byB0aGUgSFBQVCBkYXRhYmFzZS4NCg0KLS0tLS0tLS0tLS0tLS0tLS0tLS0tLS0tLS0tLS0tLS0tLS0tLS0tLS0tLS0tLS0tLS0tLS0tLS0tLS0tLS0tLS0tLS0tLS0tDQoNCjo6OiByZWZTZXQNClsxXSBPRUNELiBHdWlkZWxpbmUgTm8uIDQ5NzogRGVmaW5lZCBBcHByb2FjaGVzIG9uIFNraW4gU2Vuc2l0aXNhdGlvbi4gMjAyMS4gPGh0dHBzOi8vZG9pLm9yZy8xMC4xNzg3L2I5Mjg3OWE0LWVuPg0KDQpbMl0gT0VDRC4gQW5uZXggNDogUmVwb3J0IG9mIHRoZSBIdW1hbiBEYXRhIFN1Yi1Hcm91cCBvbiB0aGUgQ3VyYXRpb24gYW5kIEV2YWx1YXRpb24gb2YgdGhlIEh1bWFuIFJlZmVyZW5jZSBEYXRhIGFuZCB0aGUgRGVyaXZhdGlvbiBvZiBBc3NvY2lhdGVkIFN1YnN0YW5jZSBDbGFzc2ZpY2F0aW9ucy4gMjAyMS4gU2VyaWVzIG9uIFRlc3RpbmcgYW5kIEFzc2Vzc21lbnQgTm8uIDMzNi4gPGh0dHBzOi8vd3d3Lm9lY2Qub3JnL2NoZW1pY2Fsc2FmZXR5L3Rlc3Rpbmcvc2VyaWVzLXRlc3RpbmctYXNzZXNzbWVudC1wdWJsaWNhdGlvbnMtbnVtYmVyLmh0bT4NCjo6Og0KDQojIFNvdXJjZSBDb2RlDQoNClRoZSBzb3VyY2UgY29kZSBmb3IgZGVyaXZpbmcgdGhlIGNsYXNzaWZpY2F0aW9ucyBpcyBzYXZlZCBhcyBhbiBleHRlcm5hbCBSIGZpbGUuIENvbnRlbnRzIG9mIHRoZSBmaWxlIGFyZSBzaG93biBoZXJlLiBTb3VyY2luZyB0aGUgZmlsZSB3aWxsIGxvYWQgdGhlIGRwbHlyLCByZWFkciwgYW5kIHRpZHlyIHBhY2thZ2VzLiBJbiBhZGRpdGlvbiwgdGhpcyBub3RlYm9vayBjYWxscyBmdW5jdGlvbnMgZnJvbSB0aGUgcmVhZHhsIGFuZCBvcGVueGxzeCBwYWNrYWdlcy4NCg0KOjo6IHNjcm9sbC1sb25nDQoNCmBgYHtyLCBtZXNzYWdlPUZBTFNFLCB3YXJuaW5nPUZBTFNFLCBjb21tZW50ID0gIiJ9DQpzb3VyY2UoIkhQUFQtY2xhc3NpZmljYXRpb24uUiIpDQoNCiMgUHJpbnQgY29udGVudHMgb2YgZmlsZQ0KcmVhZExpbmVzKCJIUFBULWNsYXNzaWZpY2F0aW9uLlIiKSB8PiBjYXQoc2VwID0gIlxuIikNCmBgYA0KOjo6DQoNCiMgRGF0YSBDbGVhbg0KDQpGaXJzdCwgdGhlIGRhdGEgYXJlIHJlYWQgaW4gYXMgYSB0aWJibGUgd2l0aCAyLDI3NyByb3dzIGFuZCAzMSBjb2x1bW5zLg0KDQpgYGB7cn0NCmZuYW1lIDwtICIyMDIyLjA4LjEwX0hQUFRfZGF0YWJhc2V2Mi54bHN4Ig0KZGF0IDwtIHJlYWR4bDo6cmVhZF9leGNlbChmbmFtZSwgc2hlZXQgPSAxLCBjb2xfdHlwZXMgPSAidGV4dCIsIG5hID0gYygiIiwgIm5hIikpDQpkaW0oZGF0KQ0KYGBgDQoNCkZvciBkYXRhIGNsZWFuaW5nLCB3ZSB1c2UgdGhlIGZvbGxvd2luZyBwcm9jZWR1cmU6DQogIA0KMS4gIFJlbW92ZSB0ZXN0IHJlc3VsdHMgd2l0aCAqKlJSUyA9IDUqKg0KMi4gIEFzc2lnbiB1bmlxdWUgY2hlbWljYWwgSURzIGJhc2VkIG9uIENBU1JOIHdoZW4gYXZhaWxhYmxlLCBhbmQgJ1N5bm9ueW1zJyBjb2x1bW4gb3RoZXJ3aXNlLg0KMy4gIEZvciBjb2RpbmcgcHVycG9zZXMsIGNvbHVtbnMgJ0NhbGwnLCAnQ29uYy4oJSknLCAnRFNBLijOvGcvY20yKScsICdEU0ExKy4ozrxnL2NtMiknLCBhbmQgJ0RTQTA1LijOvGcvY20yKScgYXJlIHJlbmFtZWQgdG8gJ3BuJywgJ2NvbmMnLCAnZHNhJywgJ2RzYTEnLCBhbmQgJ2RzYTA1JywgcmVzcGVjdGl2ZWx5Lg0KNC4gIFRoZSBjb2x1bW5zIGNvbmMsIGRzYSwgZHNhMSwgYW5kIGRzYTA1IGFyZSBjb252ZXJ0ZWQgdG8gbnVtZXJpYy4gSWYgbXVsdGlwbGUgdmFsdWVzIGFyZSBnaXZlbiAoZS5nLiwgcmFuZ2UsIG1peHR1cmUpLCB0aGUgbWluaW11bSBudW1lcmljIHZhbHVlIGlzIHJlY29yZGVkIGFuZCB1c2VkIGZvciBjbGFzc2lmaWNhdGlvbi4NCg0KVGhlIGRhdGEgYXJlIGZpbHRlcmVkIHRvIGtlZXAgcmVjb3JkcyB3aGVyZSB0aGUgUmVsYXRpdmUgUmVsaWFiaWxpdHkgU2NvcmUgKFJSUykgXDwgNS4NCg0KYGBge3J9DQpkYXQyIDwtIGRhdCAlPiUgZmlsdGVyKFJSUyAhPSAiNSIpDQpkaW0oZGF0MikNCmBgYA0KDQoNCkhvdyBtYW55IHVuaXF1ZSwgbm9uLW1pc3NpbmcgQ0FTUk4gYXJlIGluIHRoZSBkYXRhYmFzZT8NCg0KYGBge3J9DQpkYXQyICU+JQ0KICBmaWx0ZXIoQ0FTUk4gIT0gIk5vdCBhdmFpbGFibGUiKSAlPiUNCiAgcHVsbChDQVNSTikgJT4lDQogIHVuaXF1ZSgpICU+JQ0KICBsZW5ndGgoKQ0KYGBgDQoNCkFtb25nIHRoZSBzdWJzdGFuY2VzIHdpdGhvdXQgYSBDQVNSTiwgaG93IG1hbnkgdW5pcXVlIG5hbWVzIGFyZSB0aGVyZT8NCiAgDQpgYGB7ciwgcGFnZWQucHJpbnQ9VFJVRX0NCmRhdDIgJT4lDQogIGZpbHRlcihDQVNSTiA9PSAiTm90IGF2YWlsYWJsZSIpICU+JQ0KICBwdWxsKFN5bm9ueW1zKSAlPiUNCiAgdW5pcXVlKCkgJT4lDQogIGxlbmd0aCgpDQpgYGANCg0KVGhlcmUgYXJlIDExNDggdW5pcXVlIENBU1JOcyBpbiB0aGUgZGF0YS4gQW1vbmcgcmVjb3JkcyB3aXRoIG5vIENBU1JOLCB0aGVyZSBhcmUgMjE4IHVuaXF1ZSBuYW1lcy4gVGhlIDIyNTUgdGVzdCByZXN1bHRzIHdpdGggUlJTIFw8IDUgcmVsYXRlIHRvIGEgdG90YWwgb2YgMTM2NiBzdWJzdGFuY2VzIHdpdGggYSB1bmlxdWUgSUQuDQoNClVuaXF1ZSBzdWJzdGFuY2UgSURzIGFyZSBjcmVhdGVkIGZvciBlYWNoIHN1YnN0YW5jZSB1c2luZyB0aGUgQ0FTUk4gYW5kIFN5bm9ueW1zLg0KDQpgYGB7cn0NCmRhdDIgPC0gZGF0MiAlPiUNCiAgbXV0YXRlKGNpZCA9IGlmZWxzZShDQVNSTiA9PSAiTm90IGF2YWlsYWJsZSIsIFN5bm9ueW1zLCBDQVNSTikpDQoobl9jaWQgPC0gbGVuZ3RoKHVuaXF1ZShkYXQyJGNpZCkpKQ0KYGBgDQoNClRoZSBkYXRhIGFyZSB0cmltbWVkIHRvIGtlZXAgb25seSBjb2x1bW5zIHRoYXQgYXJlIG5lZWRlZC4gQ29sdW1ucyBhcmUgcmVuYW1lZC4NCg0KYGBge3J9DQpkYXQyIDwtIGRhdDIgJT4lDQogIHNlbGVjdCgNCiAgICBhbGxfb2YoDQogICAgICBjKCJSZWNvcmQuTm8iLA0KICAgICAgICAiY2lkIiwNCiAgICAgICAgZHNhID0gIkRTQS4ozrxnL2NtMikiLA0KICAgICAgICBwbiA9ICJDYWxsIiwNCiAgICAgICAgY29uYyA9ICJDb25jLiglKSIsDQogICAgICAgIGRzYTEgPSAiRFNBMSsuKM68Zy9jbTIpIiwNCiAgICAgICAgZHNhMDUgPSAiRFNBMDUuKM68Zy9jbTIpIikpDQogICkNCmBgYA0KDQpTZXQgdXAgZXhhbXBsZSByb3cgaW5kZXgNCg0KYGBge3J9DQpzYW1wbGVDaWRzIDwtIGMoIjExMTctNTUtMSIsICIxNzg2LTA4LTkiLCAiODUtOTEtNiIsICI2OTY5LTQ5LTkiLCAiMTA2LTI0LTEiLCAiMTA2LTI2LTMiLCAiNjQtMTctNSIsICIxNTAtODYtNyIsICIxNjQwOS00My0xIiwgIjE1MC0xMy0wIiwgIjU2LTc1LTciLCAiNTAtNTMtMyIsICIyNDQyLTEwLTYiLCAiNzYzLTM1LTkiLCAiMzM3NC0yMi05IiwgIjU3Mzc4LTY4LTQiLCAiODAwMy0wNS0yIiwgIjgyMy0yMi0zIiwgIjExNi0yNi03IiwgIjMwMi0wMS0yIiwgIjYxNy01NC05IiwgIjQwNjA3LTQ4LTUiLCAiMTEyLTcyLTEiLCAiNTQ0NjQtNTctMiIsICIyODIxOS02MS02IikNCmV4YW1wbGVfcm93c19pbmRpdiA8LSBzb3J0KHdoaWNoKGRhdDIkY2lkICVpbiUgc2FtcGxlQ2lkcykpDQpleGFtcGxlX3Jvd3NfZ3JvdXAgPC0gc29ydCh3aGljaCh1bmlxdWUoZGF0MiRjaWQpICVpbiUgc2FtcGxlQ2lkcykpDQoNCmluZGl2X2NvbHMgPC0gYygiY2lkIiwgImRzYV9uZXciLCAiY29uY19uZXciLCAiZHNhMV9uZXciLCAiZHNhMDVfbmV3IiwgInBuIiwgIndvZV9zY29yZSIsICJlYyIpDQoNCmBgYA0KDQoNClRoZSBmdW5jdGlvbiByZXF1aXJlcyB0aGF0IHRoZSBmb2xsb3dpbmcgY29sdW1ucyBiZSBudW1lcmljLg0KDQotICAgQ29uY2VudHJhdGlvbiAoJSkgLSBgY29uY2ANCi0gICBEb3NlIHBlciBza2luIGFyZWEgKM68Zy9jbTxzdXA+Mjwvc3VwPikgLSBgZHNhYA0KLSAgIEh5cG90aGV0aWNhbCBkb3NlIHBlciBza2luIGFyZWEgcmVzdWx0aW5nIGluIG9uZSB0ZXN0IHN1YmplY3Qgd2l0aCBhIHBvc2l0aXZlIHRlc3QgcmVzdWx0IHVuZGVyIHRoZSBjb25kaXRpb25zIG9mIHRoZSB0ZXN0ICjOvGcvY208c3VwPjI8L3N1cD4pIC0gYGRzYTErYA0KDQpUaGUgY2xhc3NpZmljYXRpb25zIHdpbGwgYWxzbyBiZSBkZXJpdmVkIHVzaW5nIHRoZSBoeXBvdGhldGljYWwgZG9zZSBwZXIgc2tpbiBhcmVhIHJlc3VsdGluZyBpbiA1JSBpbmNpZGVuY2UgdW5kZXIgdGhlIGNvbmRpdGlvbnMgb2YgdGhlIHRlc3QgKM68Zy9jbTxzdXA+Mjwvc3VwPikgLSBgZHNhMDVgLSBpbiBwbGFjZSBvZiBgZHNhMStgLg0KDQpOdW1lcmljIGNvbnZlcnNpb24gaXMgcGVyZm9ybWVkIHVzaW5nIHRoZSBzb3VyY2VkIGZ1bmN0aW9uIGB0b19udW1gLg0KDQpgYGB7cn0NCmRhdDIkY29uY19uZXcgPC0gdG9fbnVtKGRhdDIsICJjb25jIiwNCiAgICAgICAgICAgICAgICAgICAgICAgIG5hLnZhbHVlcyA9IGMoIk5vdCBhdmFpbGFibGUiKSwNCiAgICAgICAgICAgICAgICAgICAgICAgIG1hdGNoX3BhdHRlcm4gPSAidG98YW5kIg0KKQ0KZGF0MiRkc2FfbmV3IDwtIHRvX251bShkYXQyLCAiZHNhIiwNCiAgICAgICAgICAgICAgICAgICAgICAgbmEudmFsdWVzID0gIk5vdCBhdmFpbGFibGUiLA0KICAgICAgICAgICAgICAgICAgICAgICBtYXRjaF9wYXR0ZXJuID0gIi0iDQopDQpkYXQyJGRzYTFfbmV3IDwtIHRvX251bShkYXQyLCAiZHNhMSIsDQogICAgICAgICAgICAgICAgICAgICAgICBuYS52YWx1ZXMgPSBjKCJOb3QgYXBwbGljYWJsZSIsICJOb3QgYXZhaWxhYmxlIiksDQogICAgICAgICAgICAgICAgICAgICAgICBtYXRjaF9wYXR0ZXJuID0gInRvIg0KKQ0KZGF0MiRkc2EwNV9uZXcgPC0gdG9fbnVtKGRhdDIsICJkc2EwNSIsDQogICAgICAgICAgICAgICAgICAgICAgICAgbmEudmFsdWVzID0gYygiTm90IGFwcGxpY2FibGUiLCAiTm90IGF2YWlsYWJsZSIpLA0KICAgICAgICAgICAgICAgICAgICAgICAgIG1hdGNoX3BhdHRlcm4gPSAidG8iDQopDQpgYGANCg0KDQojIENsYXNzaWZpY2F0aW9uDQoNCiMjIEluZGl2aWR1YWwgTWV0aG9kcw0KDQpXZSB1c2UgdGhlIGZ1bmN0aW9uIHNvdXJjZWQgYGhwcHRfZ2hzX2NsYXNzYCB0byBkZXJpdmUgR0hTIGNsYXNzaWZpY2F0aW9ucyB1c2luZyAzIG1ldGhvZHM6DQogIA0KMS4gIFdlaWdodC1vZi1FdmlkZW5jZSBTY29yZSAoV29FKQ0KMi4gIE1lZGlhbi1MaWtlIExvY2F0aW9uIFBhcmFtZXRlciAoTUxMUCkNCjMuICBNZWRpYW4gU2Vuc2l0aXphdGlvbiBQb3RlbmN5IEVzdGltYXRlIChNU1BFKQ0KDQpUaGUgZnVuY3Rpb24gYWxzbyByZXR1cm5zIHRoZSBpbmRpdmlkdWFsIGV4dHJhcG9sYXRlZCBjbGFzc2lmaWNhdGlvbnMgdXNlZCB0byBkZXJpdmUgdGhlIEdIUyBjbGFzc2lmaWNhdGlvbnMuDQoNCiMjIyBEU0ExKyANCg0KYGBge3J9DQpkc2ExX2NsYXNzIDwtIGhwcHRfZ2hzX2NsYXNzKA0KICBkdCA9IGRhdDIsDQogIGNhbGxfY29sID0gcG4sICMgY29sdW1uIGNvbnRhaW5pbmcgYWN0aXZlL2luYWN0aXZlIGNhbGxzDQogIGNvbmNfY29sID0gY29uY19uZXcsDQogIGRzYV9jb2wgPSBkc2FfbmV3LA0KICBkc2ExX2NvbCA9IGRzYTFfbmV3LA0KICBncm91cF9jb2wgPSBjaWQsDQogIGluYWN0aXZlX25hbWUgPSAiSW5hY3RpdmUiLA0KICBhY3RpdmVfbmFtZSA9ICJBY3RpdmUiDQopDQpgYGANCg0KVGhlIGZpcnN0IGxpc3QgaXRlbSBvZiB0aGUgb3V0cHV0IGNvbnRhaW5zIHRoZSByZXN1bHRzIGZvciBlYWNoIGluZGl2aWR1YWwgbWV0aG9kIGZvciBlYWNoIG9mIHRoZSBjaGVtaWNhbHMuIFRoZSBXZWlnaHQgb2YgRXZpZGVuY2UgU2NvcmUsIE1MTFAsIGFuZCBNU1BFIHVzZSB0aGUgaW5kaXZpZHVhbCBFQ3MgdG8gY2xhc3NpZnkgR0hTIGNhdGVnb3JpZXMuIA0KDQpgYGB7cn0NCmRzYTFfY2xhc3Mkb3ZlcmFsbF9jbGFzc2VzW2V4YW1wbGVfcm93c19ncm91cCxdDQpgYGANCg0KVGhlIHNlY29uZCBsaXN0IGl0ZW0gb2YgdGhlIG91dHB1dCBjb250YWlucyB0aGUgaW5kaXZpZHVhbCByZWNvcmQgV29FIHNjb3JlcyBhbmQgdGhlIEVDcyBmb3IgZWFjaCByZWNvcmQuIA0KDQpgYGB7cn0NCmRzYTFfY2xhc3MkZWNfaW5kaXZbZXhhbXBsZV9yb3dzX2luZGl2LGluZGl2X2NvbHNdDQpgYGANCg0KIyMjIERTQTUlDQoNCkNsYXNzaWZpY2F0aW9ucyBhcmUgZGVyaXZlZCB1c2luZyB0aGUgRFNBMDUgdmFsdWUgaW4gcGxhY2Ugb2YgRFNBMSsuDQoNCmBgYHtyfQ0KZHNhMDVfY2xhc3MgPC0gaHBwdF9naHNfY2xhc3MoDQogIGR0ID0gZGF0MiwNCiAgY2FsbF9jb2wgPSBwbiwgIyBjb2x1bW4gY29udGFpbmluZyBhY3RpdmUvaW5hY3RpdmUgY2FsbHMNCiAgY29uY19jb2wgPSBjb25jX25ldywNCiAgZHNhX2NvbCA9IGRzYV9uZXcsDQogIGRzYTFfY29sID0gZHNhMDVfbmV3LA0KICBncm91cF9jb2wgPSBjaWQsDQogIGluYWN0aXZlX25hbWUgPSAiSW5hY3RpdmUiLA0KICBhY3RpdmVfbmFtZSA9ICJBY3RpdmUiDQopDQpgYGANCg0KYGBge3J9DQpkc2EwNV9jbGFzcyRvdmVyYWxsX2NsYXNzZXNbZXhhbXBsZV9yb3dzX2dyb3VwLF0NCmBgYA0KDQpgYGB7cn0NCmRzYTA1X2NsYXNzJGVjX2luZGl2W2V4YW1wbGVfcm93c19pbmRpdixpbmRpdl9jb2xzXQ0KYGBgDQoNCiMjIE92ZXJhbGwgV2VpZ2h0IG9mIEV2aWRlbmNlDQoNCldlIHVzZSB0aGUgZnVuY3Rpb24gYGhwcHRfb3ZlcmFsbF9jbGFzc2lmaWNhdGlvbmAgdG8gYXNzaWduIG92ZXJhbGwgd2VpZ2h0IG9mIGV2aWRlbmNlIEdIUyBjbGFzaWZpY2F0aW9ucy4gSWYgdGhlIE1MTFAsIE1TUEUsIGFuZCBXb0UgU2NvcmUgb3V0Y29tZXMgYXJlIGNvbmNvcmRhbnQsIHRoZW4gdGhhdCBpcyB0aGUgb3ZlcmFsbCBHSFMgY2xhc3NpZmljYXRpb24uICANCg0KIyMjIERTQTErDQoNCkZvciBlYWNoIGNoZW1pY2FsLCB0aGUgb3V0cHV0IHNob3dzIHRoZSBpbmRpdmlkdWFsIG1ldGhvZCBvdXRjb21lcywgYSBzdW1tYXJ5IG9mIHRoZSBpbmRpdmlkdWFsIEVDcywgYW5kIHRoZSBvdmVyYWxsIEdIUyBjbGFzc2lmaWNhdGlvbnMuIA0KDQpgYGB7cn0NCmRzYTFfb3ZlcmFsbCA8LSBocHB0X292ZXJhbGxfY2xhc3NpZmljYXRpb24oaHBwdF9naHNfY2xhc3NfbGlzdCA9IGRzYTFfY2xhc3MsIGdyb3VwX2NvbCA9IGNpZCkNCmRzYTFfb3ZlcmFsbFtleGFtcGxlX3Jvd3NfZ3JvdXAsXQ0KYGBgDQoNCiMjIyBEU0E1JSANCg0KYGBge3J9DQpkc2EwNV9vdmVyYWxsIDwtIGhwcHRfb3ZlcmFsbF9jbGFzc2lmaWNhdGlvbihkc2EwNV9jbGFzcywgY2lkKQ0KZHNhMDVfb3ZlcmFsbFtleGFtcGxlX3Jvd3NfZ3JvdXAsXQ0KYGBgDQoNCg0KIyMjIFJlc29sdmluZyBEaXNjb3JkYW5jZQ0KDQpXaGVuIHRoZSB0aHJlZSBhcHByb2FjaGVzIGRpc2FncmVlIGZvciB0aGUgR0hTIG91dGNvbWVzLCBgaHBwdF9vdmVyYWxsX2NsYXNzaWZpY2F0aW9uYCBkb2VzIG5vdCBhc3NpZ24gYSB2YWx1ZS4gRm9yIHRoZXNlIGNoZW1pY2FscywgdGhlIE9FQ0QgRUcgREFTUyBldmFsdWF0ZWQgdGhlIHRlc3QgcmVzdWx0cyBhbmQgYXBwbGllZCBydWxlLWd1aWRlZCBleHBlcnQganVkZ2VtZW50IHRvIGRlZmluZSBhIGNvbnNlbnN1cyBmb3IgR0hTPHN1Yj5TVUI8L3N1Yj4gYW5kIEdIUzxzdWI+Qk9SREVSPC9zdWI+LiBUaGUgZnVuY3Rpb24gYGhwcHRfcmVzb2x2ZV9vdmVyYWxsYCBhcHBsaWVzIHRoZXNlIHJ1bGVzIHdoZXJlIGFwcGxpY2FibGUuIA0KDQpTYXZlIHRoZSB2YWx1ZXMgdG8gY29tcGFyZSBiZWZvcmUgYW5kIGFmdGVyOg0KDQpgYGB7cn0NCnN1Yl9jb2xzIDwtIGMoIndvZV9naHNfc3ViIiwgIm1sbHBfZ2hzX3N1YiIsICJtc3BlX2doc19zdWIiKQ0KDQpkc2ExX3N1Yl9kaXNjb3JkIDwtIGFwcGx5KGRzYTFfb3ZlcmFsbFtzdWJfY29sc10sIDEsIGZ1bmN0aW9uKHgpIGxlbmd0aCh1bmlxdWUobmEub21pdCh4KSkpID4gMSkNCmRzYTFfc3ViX2Rpc2NvcmRfZGF0YSA8LSBkc2ExX292ZXJhbGxbZHNhMV9zdWJfZGlzY29yZCxdDQoNCmRzYTA1X3N1Yl9kaXNjb3JkIDwtIGFwcGx5KGRzYTA1X292ZXJhbGxbc3ViX2NvbHNdLCAxLCBmdW5jdGlvbih4KSBsZW5ndGgodW5pcXVlKG5hLm9taXQoeCkpKSA+IDEpDQpkc2EwNV9zdWJfZGlzY29yZF9kYXRhIDwtIGRzYTA1X292ZXJhbGxbZHNhMDVfc3ViX2Rpc2NvcmQsXQ0KDQpib3JkZXJfY29scyA8LSBjKCJ3b2VfZ2hzX2JvcmRlciIsICJtbGxwX2doc19ib3JkZXIiLCAibXNwZV9naHNfYm9yZGVyIikNCmRzYTFfYm9yZGVyX2Rpc2NvcmQgPC0gYXBwbHkoZHNhMV9vdmVyYWxsW2JvcmRlcl9jb2xzXSwgMSwgZnVuY3Rpb24oeCkgbGVuZ3RoKHVuaXF1ZShuYS5vbWl0KHgpKSkgPiAxKQ0KZHNhMV9ib3JkZXJfZGlzY29yZF9kYXRhIDwtIGRzYTFfb3ZlcmFsbFtkc2ExX2JvcmRlcl9kaXNjb3JkLF0NCg0KZHNhMDVfYm9yZGVyX2Rpc2NvcmQgPC0gYXBwbHkoZHNhMDVfb3ZlcmFsbFtib3JkZXJfY29sc10sIDEsIGZ1bmN0aW9uKHgpIGxlbmd0aCh1bmlxdWUobmEub21pdCh4KSkpID4gMSkNCmRzYTA1X2JvcmRlcl9kaXNjb3JkX2RhdGEgPC0gZHNhMDVfb3ZlcmFsbFtkc2EwNV9ib3JkZXJfZGlzY29yZCxdDQpgYGANCg0KYGBge3J9DQpyZXNvbHZlX291dCA8LSBocHB0X3Jlc29sdmVfb3ZlcmFsbCgNCiAgZHNhMV9vdmVyYWxsID0gZHNhMV9vdmVyYWxsLA0KICBkc2EwNV9vdmVyYWxsID0gZHNhMDVfb3ZlcmFsbCwNCiAgZ3JvdXBfY29sID0gImNpZCINCikNCmRzYTFfb3ZlcmFsbCA8LSByZXNvbHZlX291dCRkc2ExX292ZXJhbGwNCmRzYTA1X292ZXJhbGwgPC0gcmVzb2x2ZV9vdXQkZHNhMDVfb3ZlcmFsbA0KYGBgDQoNCiMjIyMgR0hTIFNVQg0KDQojIyMjIyBEU0ExKw0KDQpPcmlnaW5hbDogDQoNCmBgYHtyfQ0Kc3ViX2NvbHMgPC0gYygiY2lkIiwgIm92ZXJhbGxfZ2hzX3N1YiIsICJtbGxwX2doc19zdWIiLCAibXNwZV9naHNfc3ViIiwgIndvZV9naHNfc3ViIiwgIjFBLSIsICIxQSIpDQpkc2ExX3N1Yl9kaXNjb3JkX2RhdGFbLHN1Yl9jb2xzXQ0KYGBgDQoNClJ1bGUgQmFzZWQ6IA0KDQpgYGB7cn0NCmRzYTFfb3ZlcmFsbFtkc2ExX3N1Yl9kaXNjb3JkLHN1Yl9jb2xzXQ0KYGBgDQoNCiMjIyMjIERTQTUlDQoNCk9yaWdpbmFsOiANCg0KYGBge3J9DQpkc2EwNV9zdWJfZGlzY29yZF9kYXRhWyxzdWJfY29sc10NCmBgYA0KDQpSdWxlIEJhc2VkOiANCg0KYGBge3J9DQpkc2EwNV9vdmVyYWxsW2RzYTA1X3N1Yl9kaXNjb3JkLHN1Yl9jb2xzXQ0KYGBgDQoNCiMjIyMgR0hTIEJPUkRFUg0KDQojIyMjIyBEU0ExKw0KDQpPcmlnaW5hbDogDQoNCmBgYHtyfQ0KYm9yZGVyX2NvbHMgPC0gYygiY2lkIiwgIm92ZXJhbGxfZ2hzX2JvcmRlciIsICJtbGxwX2doc19ib3JkZXIiLCAibXNwZV9naHNfYm9yZGVyIiwgIndvZV9naHNfYm9yZGVyIiwgIjFBLSIsICIxQSIpDQpkc2ExX2JvcmRlcl9kaXNjb3JkX2RhdGFbLGJvcmRlcl9jb2xzXQ0KYGBgDQoNClJ1bGUgQmFzZWQ6IA0KDQpgYGB7cn0NCmRzYTFfb3ZlcmFsbFtkc2ExX2JvcmRlcl9kaXNjb3JkLGJvcmRlcl9jb2xzXQ0KYGBgDQoNCg0KIyMjIyMgRFNBNSUNCg0KT3JpZ2luYWw6IA0KDQpgYGB7cn0NCmRzYTA1X2JvcmRlcl9kaXNjb3JkX2RhdGFbLGJvcmRlcl9jb2xzXQ0KYGBgDQoNClJ1bGUgQmFzZWQ6IA0KDQpgYGB7cn0NCmRzYTA1X292ZXJhbGxbZHNhMDVfYm9yZGVyX2Rpc2NvcmQsYm9yZGVyX2NvbHNdDQpgYGANCiMgUmVwcm9kdWNpYmlsaXR5DQoNCiMjIEdIUyBCSU4NCg0KIyMjIERTQTErDQoNCmBgYHtyfQ0KZHNhMV9yZXBybyA8LSBocHB0X3JlcHJvKGRzYTFfb3ZlcmFsbCwgY2lkKQ0KZHNhMV9yZXByb1tleGFtcGxlX3Jvd3NfZ3JvdXAsYygiY2lkIiwgIm92ZXJhbGxfZ2hzX2JpbiIsICJyZXByb19naHNfYmluIiwgInRvdGFsX3Rlc3RzX3JlcHJvX2JpbiIsICJOQyIsICIxQiIsICIxQisiLCAiUE9TIiwgIjFBLSIsICIxQSIpXSAlPiUNCiAgZmlsdGVyKCFpcy5uYShyZXByb19naHNfYmluKSkNCmBgYA0KDQojIyMgRFNBNSUNCg0KYGBge3J9DQpkc2EwNV9yZXBybyA8LSBocHB0X3JlcHJvKGRzYTA1X292ZXJhbGwsIGNpZCkNCmRzYTA1X3JlcHJvW2V4YW1wbGVfcm93c19ncm91cCxjKCJjaWQiLCAib3ZlcmFsbF9naHNfYmluIiwgInJlcHJvX2doc19iaW4iLCAidG90YWxfdGVzdHNfcmVwcm9fYmluIiwgIk5DIiwgIjFCIiwgIjFCKyIsICJQT1MiLCAiMUEtIiwgIjFBIildICU+JQ0KICBmaWx0ZXIoIWlzLm5hKHJlcHJvX2doc19iaW4pKQ0KYGBgDQoNCiMjIEdIUyBTVUINCg0KIyMjIERTQTErDQoNCmBgYHtyfQ0KZHNhMV9yZXByb1tleGFtcGxlX3Jvd3NfZ3JvdXAsYygiY2lkIiwgIm92ZXJhbGxfZ2hzX3N1YiIsICJyZXByb19naHNfc3ViIiwgInRvdGFsX3Rlc3RzX3JlcHJvX3N1YiIsICJOQyIsICIxQiIsICIxQisiLCAiMUEtIiwgIjFBIildICU+JQ0KICBmaWx0ZXIoIWlzLm5hKHJlcHJvX2doc19zdWIpKQ0KYGBgDQoNCiMjIyBEU0E1JQ0KDQpgYGB7cn0NCmRzYTA1X3JlcHJvW2V4YW1wbGVfcm93c19ncm91cCxjKCJjaWQiLCAib3ZlcmFsbF9naHNfc3ViIiwgInJlcHJvX2doc19zdWIiLCAidG90YWxfdGVzdHNfcmVwcm9fc3ViIiwgIk5DIiwgIjFCIiwgIjFCKyIsICIxQS0iLCAiMUEiKV0gJT4lDQogIGZpbHRlcighaXMubmEocmVwcm9fZ2hzX3N1YikpDQpgYGANCg0KIyBFeGFtcGxlOiBQZW5pY2lsbGluIEcgKENBU1JOIDYxLTMzLTYvRUMgMjAwLTUwNi0zKQ0KDQojIyBEU0ExKw0KDQpEaXN0cmlidXRpb24gb2YgaW5kaXZpZHVhbCBleHRyYXBvbGF0ZWQgY2xhc3NpZmljYXRpb25zIGJhc2VkIG9uIERTQTErOg0KDQpgYGB7cn0NCmRzYTFfY2xhc3MkZWNfaW5kaXYgJT4lIA0KICBmaWx0ZXIoY2lkID09ICI2MS0zMy02IikgJT4lDQogIGdyb3VwX2J5KGVjKSAlPiUNCiAgdGFsbHkoKQ0KYGBgDQoNClJlc3VsdHMgZnJvbSBpbmRpdmlkdWFsIG1ldGhvZHM6DQoNCmBgYHtyfQ0KZHNhMV9jbGFzcyRvdmVyYWxsX2NsYXNzZXMgJT4lDQogIGZpbHRlcihjaWQgPT0gIjYxLTMzLTYiKSAlPiUNCiAgcGl2b3RfbG9uZ2VyKGNvbHMgPSAtY2lkKQ0KYGBgDQoNCk92ZXJhbGwgd2VpZ2h0IG9mIGV2aWRlbmNlIGNsYXNzaWZpY2F0aW9uIGFuZCByZXByb2R1Y2liaWxpdHk6DQoNCmBgYHtyfQ0KZHNhMV9yZXBybyAlPiUNCiAgZmlsdGVyKGNpZCA9PSAiNjEtMzMtNiIpICU+JQ0KICBzZWxlY3QoY2lkLCBzdGFydHNfd2l0aCgib3ZlcmFsbCIpLCBzdGFydHNfd2l0aCgicmVwcm8iKSkgJT4lDQogIHBpdm90X2xvbmdlcihjb2xzID0gLWNpZCwgdmFsdWVzX3RyYW5zZm9ybSA9IGFzLmNoYXJhY3RlcikNCmBgYA0KDQojIyBEU0E1JQ0KDQpEaXN0cmlidXRpb24gb2YgaW5kaXZpZHVhbCBleHRyYXBvbGF0ZWQgY2xhc3NpZmljYXRpb25zIGJhc2VkIG9uIERTQTUlOg0KDQpgYGB7cn0NCmRzYTA1X2NsYXNzJGVjX2luZGl2ICU+JSANCiAgZmlsdGVyKGNpZCA9PSAiNjEtMzMtNiIpICU+JQ0KICBncm91cF9ieShlYykgJT4lDQogIHRhbGx5KCkNCmBgYA0KDQpSZXN1bHRzIGZyb20gaW5kaXZpZHVhbCBtZXRob2RzOg0KDQpgYGB7cn0NCmRzYTA1X2NsYXNzJG92ZXJhbGxfY2xhc3NlcyAlPiUNCiAgZmlsdGVyKGNpZCA9PSAiNjEtMzMtNiIpICU+JQ0KICBwaXZvdF9sb25nZXIoY29scyA9IC1jaWQpDQpgYGANCg0KT3ZlcmFsbCB3ZWlnaHQgb2YgZXZpZGVuY2UgY2xhc3NpZmljYXRpb24gYW5kIHJlcHJvZHVjaWJpbGl0eToNCg0KYGBge3J9DQpkc2EwNV9yZXBybyAlPiUNCiAgZmlsdGVyKGNpZCA9PSAiNjEtMzMtNiIpICU+JQ0KICBzZWxlY3QoY2lkLCBzdGFydHNfd2l0aCgib3ZlcmFsbCIpLCBzdGFydHNfd2l0aCgicmVwcm8iKSkgJT4lDQogIHBpdm90X2xvbmdlcihjb2xzID0gLWNpZCwgdmFsdWVzX3RyYW5zZm9ybSA9IGFzLmNoYXJhY3RlcikNCmBgYA0KDQojIFNhdmUgT3V0cHV0DQoNCkFkZCBpZGVudGlmaWVycyBiYWNrLg0KDQpgYGB7cn0NCmRpY3QgPC0gZnVsbF9qb2luKA0KICBzZWxlY3QoZGF0LCBSZWNvcmQuTm8sIFN5bm9ueW1zLCBDQVNSTiwgRUMuTm8pLA0KICBzZWxlY3QoZGF0MiwgUmVjb3JkLk5vLCBjaWQpLA0KICBieSA9ICJSZWNvcmQuTm8iDQopDQoNCmRzYTFfY2xhc3MkZWNfaW5kaXYgPC0gcmlnaHRfam9pbigNCiAgZGljdCwgZHNhMV9jbGFzcyRlY19pbmRpdiwgYnkgPSBjKCJjaWQiLCAiUmVjb3JkLk5vIikNCikgJT4lDQogIHNlbGVjdCgtY2lkKQ0KDQpkc2EwNV9jbGFzcyRlY19pbmRpdiA8LSByaWdodF9qb2luKA0KICBkaWN0LCBkc2EwNV9jbGFzcyRlY19pbmRpdiwgYnkgPSBjKCJjaWQiLCAiUmVjb3JkLk5vIikpICU+JQ0KICBzZWxlY3QoLWNpZCkNCg0KZGljdCA8LSBkaWN0ICU+JQ0KICBzZWxlY3QoLVJlY29yZC5ObykgJT4lDQogIGRpc3RpbmN0KCkgJT4lDQogIGdyb3VwX2J5KENBU1JOLCBFQy5ObywgY2lkKSAlPiUNCiAgbXV0YXRlKFN5bm9ueW1zID0gZ3N1YigiW1s6Y250cmw6XV0rIiwgIiAiLCBTeW5vbnltcykpICU+JQ0KICBzdW1tYXJpemUoU3lub255bXMgPSBwYXN0ZSh1bmlxdWUodW5saXN0KHN0cnNwbGl0KFN5bm9ueW1zLCAiOyAiKSkpLCBjb2xsYXBzZSA9ICI7ICIpLA0KICAgICAgICAgICAgLmdyb3VwcyA9ICJrZWVwIikgJT4lDQogIHVuZ3JvdXAoKSAlPiUNCiAgcmVsb2NhdGUoU3lub255bXMpDQoNCmRzYTFfY2xhc3Mkb3ZlcmFsbF9jbGFzc2VzIDwtIHJpZ2h0X2pvaW4oZGljdCwgZHNhMV9jbGFzcyRvdmVyYWxsX2NsYXNzZXMsIGJ5ID0gImNpZCIpICU+JQ0KICBzZWxlY3QoLWNpZCkNCmRzYTFfcmVwcm8gPC0gcmlnaHRfam9pbihkaWN0LCBkc2ExX3JlcHJvLCBieSA9ICJjaWQiKSAlPiUgc2VsZWN0KC1jaWQpDQpkc2EwNV9jbGFzcyRvdmVyYWxsX2NsYXNzZXMgPC0gcmlnaHRfam9pbihkaWN0LCBkc2EwNV9jbGFzcyRvdmVyYWxsX2NsYXNzZXMsIGJ5ID0gImNpZCIpICU+JQ0KICBzZWxlY3QoLWNpZCkNCmRzYTA1X3JlcHJvIDwtIHJpZ2h0X2pvaW4oZGljdCwgZHNhMDVfcmVwcm8sIGJ5ID0gImNpZCIpICU+JSBzZWxlY3QoLWNpZCkNCmBgYA0KDQpgYGB7cn0NCmxpYnJhcnkob3Blbnhsc3gpDQpmbmFtZTEgPC0gcGFzdGUwKGZvcm1hdChTeXMudGltZSgpLCAiJVklbSVkLSVIJU0tIiksICItaHBwdENsYXNzLURTQTFwbHVzLnhsc3giKQ0Kd2IgPC0gY3JlYXRlV29ya2Jvb2soKQ0KYWRkV29ya3NoZWV0KHdiLCBzaGVldE5hbWUgPSAiSW5kaXZpZHVhbEVDcyIpDQp3cml0ZURhdGEod2IsICJJbmRpdmlkdWFsRUNzIiwgZHNhMV9jbGFzcyRlY19pbmRpdiwga2VlcE5BID0gVCwgbmEuc3RyaW5nID0gIm5hIikNCmFkZFdvcmtzaGVldCh3Yiwgc2hlZXROYW1lID0gIkdIU0NsYXNzUHJlZCIpDQp3cml0ZURhdGEod2IsICJHSFNDbGFzc1ByZWQiLCBkc2ExX2NsYXNzJG92ZXJhbGxfY2xhc3Nlcywga2VlcE5BID0gVCwgbmEuc3RyaW5nID0gIm5hIikNCmFkZFdvcmtzaGVldCh3Yiwgc2hlZXROYW1lID0gIlJlcHJvZHVjaWJpbGl0eSIpDQp3cml0ZURhdGEod2IsICJSZXByb2R1Y2liaWxpdHkiLCBkc2ExX3JlcHJvLCBrZWVwTkEgPSBULCBuYS5zdHJpbmcgPSAibmEiKQ0Kc2F2ZVdvcmtib29rKHdiLCBmbmFtZTEpDQoNCmZuYW1lMiA8LSBwYXN0ZTAoZm9ybWF0KFN5cy50aW1lKCksICIlWSVtJWQtJUglTS0iKSwgIi1ocHB0Q2xhc3MtRFNBNXBlcmMueGxzeCIpDQp3YiA8LSBjcmVhdGVXb3JrYm9vaygpDQphZGRXb3Jrc2hlZXQod2IsIHNoZWV0TmFtZSA9ICJJbmRpdmlkdWFsRUNzIikNCndyaXRlRGF0YSh3YiwgIkluZGl2aWR1YWxFQ3MiLCBkc2EwNV9jbGFzcyRlY19pbmRpdiwga2VlcE5BID0gVCwgbmEuc3RyaW5nID0gIm5hIikNCmFkZFdvcmtzaGVldCh3Yiwgc2hlZXROYW1lID0gIkdIU0NsYXNzUHJlZCIpDQp3cml0ZURhdGEod2IsICJHSFNDbGFzc1ByZWQiLCBkc2EwNV9jbGFzcyRvdmVyYWxsX2NsYXNzZXMsIGtlZXBOQSA9IFQsIG5hLnN0cmluZyA9ICJuYSIpDQphZGRXb3Jrc2hlZXQod2IsIHNoZWV0TmFtZSA9ICJSZXByb2R1Y2liaWxpdHkiKQ0Kd3JpdGVEYXRhKHdiLCAiUmVwcm9kdWNpYmlsaXR5IiwgZHNhMDVfcmVwcm8sIGtlZXBOQSA9IFQsIG5hLnN0cmluZyA9ICJuYSIpDQpzYXZlV29ya2Jvb2sod2IsIGZuYW1lMikNCmBgYA0KDQojIFNlc3Npb24gSW5mbw0KDQpgYGB7cn0NCnNlc3Npb25JbmZvKCkNCmBgYA0K
